# Intrapleural hemocoagulase Bothrops atrox and early outcomes after VATS for stage IA non-small cell lung cancer

**DOI:** 10.3389/fmed.2026.1774067

**Published:** 2026-04-10

**Authors:** Yingding Ruan, Hongsheng Xue, Wenjun Cao, Chuan Long, Aiming Yang, Jianwei Han, Jiajia Yang, Zhilong Zhao

**Affiliations:** 1 Department of Thoracic Surgery, The First People’s Hospital of Jiande, Jiande, China; 2 Department of Thoracic Surgery, Affiliated Zhongshan Hospital of Dalian University, Dalian, China; 3 Department of Pharmacy, The First People’s Hospital of Jiande, Jiande, China

**Keywords:** coagulation profiles, hemocoagulase Bothrops atrox, inverse probability of treatment weighting, non-small cell lung cancer, video-assisted thoracoscopic surgery

## Abstract

**Background:**

Hemocoagulase Bothrops atrox (HBA) is used to reduce surgical bleeding, but its impact on postoperative coagulation after video-assisted thoracoscopic surgery (VATS) remains unclear. We evaluated the effects of intrapleural HBA on coagulation profiles and early recovery in stage IA non-small cell lung cancer (NSCLC).

**Methods:**

We retrospectively analyzed 442 stage IA NSCLC patients undergoing VATS, allocated to HBA (*n* = 92) or non-HBA (*n* = 350) groups. The primary outcome was postoperative coagulation; secondary outcomes were length of stay and postoperative complications. Baseline characteristics were balanced using inverse probability of treatment weighting (IPTW), followed by multivariable analyses.

**Results:**

After IPTW adjustment, intrapleural HBA was associated with a longer prothrombin time (PT; 12.83 ± 0.99 vs. 12.31 ± 1.50 s; *β* = 0.28, 95% CI 0.11–0.45, *p* = 0.001) and lower fibrinogen (FIB) levels (322.06 ± 96.68 vs. 353.52 ± 122.65 mg/dL; *β* = −33.33, 95% CI − 48.46 to −18.20, *p* < 0.001), while activated partial thromboplastin time and thrombin time did not differ significantly. In the IPTW-weighted cohort, HBA use was associated with a lower incidence of postoperative complications (6.5% vs. 12.0%; OR = 0.35, 95% CI 0.19–0.63, *p* = 0.001) and a shorter postoperative hospital stay (*β* = −2.06, 95% CI − 2.61 to −1.50, *p* < 0.001).

**Conclusion:**

Intrapleural HBA injection in stage IA NSCLC undergoing VATS is associated with modest alterations in coagulation (prolonged PT and reduced FIB) and improved early outcomes, including fewer complications and shorter hospitalization. Prospective studies are warranted to confirm these findings.

## Introduction

Lung cancer remains the leading cause of cancer-related morbidity and mortality worldwide. For patients with early-stage non–small cell lung cancer (NSCLC), anatomical lung resection continues to represent the standard curative treatment strategy (1, 2). The widespread adoption of video-assisted thoracoscopic surgery (VATS) has transformed thoracic surgical practice by minimizing access trauma, reducing postoperative pain, and facilitating earlier mobilization and discharge (3, 4). However, perioperative bleeding continues to be a frequent and clinically important problem, contributing to postoperative complications, prolonged hospitalization, and increased healthcare resource utilization (5). Even apparently modest volumes of intraoperative blood loss may still adversely influence early recovery and potentially affect longer-term oncological outcomes (6).

Against this backdrop, hemocoagulase Bothrops atrox (HBA), a hemocoagulant derived from Bothrops atrox snake venom, has attracted interest as an adjunct to improve surgical hemostasis. By promoting fibrin formation at sites of vascular injury, HBA can enhance local clot stability while exerting relatively little effect on the systemic coagulation profile (7). Experience from other surgical fields suggests that both topical and systemic administration of HBA may reduce oozing from raw surfaces and limit postoperative drainage; nevertheless, data specific to thoracic procedures—and in particular to intrapleural application during VATS—remain limited. The use of intrapleural HBA during minimally invasive lung resection therefore represents a potentially valuable yet insufficiently characterized therapeutic strategy.

Critically, the specific impact of intrapleural HBA on the postoperative coagulation cascade remains elusive. It is unknown whether this localized administration elicits measurable and clinically relevant changes in standard coagulation parameters, such as prothrombin time (PT), activated partial thromboplastin time (APTT), and fibrinogen (FIB) levels. Furthermore, a comprehensive understanding of how these potential coagulation alterations might translate into tangible clinical benefits—specifically, a reduction in complication rates and a shortening of the postoperative hospital stay—is currently lacking. This knowledge gap impedes the formulation of evidence-based guidelines for HBA use in thoracic surgical practice.

Therefore, to bridge this gap in clinical evidence, we conducted this retrospective cohort study. We aimed to rigorously evaluate the efficacy and safety of intrapleural HBA injection in patients with stage IA NSCLC undergoing VATS. Our primary objective was to delineate its effects on postoperative coagulation profiles, while secondary objectives focused on assessing its association with key recovery metrics, including postoperative complications and the duration of hospital stay.

Building on a prior, smaller matched analysis from our group (8), the current study was designed to validate and extend those observations in an expanded cohort using an inverse probability of treatment weighting (IPTW) framework and enhanced recovery after surgery (ERAS)-relevant recovery endpoints.

## Methods

### Study design

This single-center, retrospective cohort study was conducted in compliance with the Declaration of Helsinki and received approval from the Ethics Committee of The First People’s Hospital of Jiande (Approval Number: 20251124-KY-001-01). The requirement for informed consent was waived due to the retrospective nature of the study.

We consecutively screened 566 patients with NSCLC who underwent VATS at our institution between December 2021 and March 2025. The inclusion criteria were: (1) patients who received VATS; and (2) had a postoperative pathological diagnosis of stage IA NSCLC. The exclusion criteria were as follows: (1) previous chest surgery; (2) benign or metastatic lung tumor; (3) any neoadjuvant therapy; (4) reoperation within one month; (5) transfer to another hospital; (6) history of hepatitis B, cirrhosis, or splenectomy; (7) missing postoperative coagulation function tests within 1 day after surgery; and (8) age under 18 years. The final cohort comprised 442 patients, who were categorized into an HBA group (*n* = 92) and a non-HBA group (*n* = 350) based on whether they received intrapleural hemocoagulase Bothrops atrox injection. The tumor, node, and metastasis (TNM) classification was determined according to the International Association for the Study of Lung Cancer (IASLC) guidelines (9).

### Hemocoagulase Bothrops atrox

HBA is a haemostatic enzyme preparation purified from the venom of Bothrops atrox (10). Its principal action is to cleave fibrinogen, generating fibrin monomers and fibrinopeptides that rapidly assemble into stable fibrin clots (11–13). When used in appropriate doses, HBA activity is largely confined to areas of tissue injury, with minimal effect on intact vascular endothelium, and available evidence does not indicate a substantial increase in the risk of perioperative thrombosis or disseminated intravascular coagulation (10, 14–16).

In the HBA group, a solution containing 10 units of HBA diluted in 5 mL of 0.9% normal saline was prepared immediately before administration. After completion of the lung resection and confirmation of hemostasis, but prior to final chest closure, this solution was gently instilled over the staple lines and exposed pleural surfaces under direct thoracoscopic visualization. Delivery was performed using a sterile syringe connected to a long extension line introduced through the VATS access port.

Intrapleural hemocoagulase Bothrops atrox was administered as a fixed dose according to routine clinical practice and institutional experience, consistent with our previous clinical use of this approach (8). Given the absence of standardized dosing guidelines for intrapleural use, the dose was not adjusted according to patient body weight and was applied uniformly across all patients.

### Data collection and outcomes

Patient data were retrospectively collected from electronic medical records by two independent investigators, with verification by a third author to ensure accuracy. The collected variables included:

*Demographics and clinicopathologic data*: sex, age, body mass index (BMI), smoking history, comorbidities (hypertension, diabetes, coronary heart disease, chronic obstructive pulmonary disease), pathological types, and imaging description (categorized as ground glass nodule, mixed nodule, or solid nodule).

*Surgical and perioperative details*: surgical approach (uniportal video-assisted thoracoscopic surgery [U-VATS] or multiportal video-assisted thoracoscopic surgery [M-VATS]), resection site and type, number of mediastinal lymph nodes retrieved and stations explored, surgical duration, intraoperative bleeding volume, drainage time and volume, postoperative hospital stay, and postoperative complications.

*Laboratory parameters*: Preoperative and postoperative (postoperative day 1) blood samples were obtained to measure albumin (ALB) and coagulation profiles, including D-Dimer, international normalized ratio (INR), activated partial thromboplastin time (APTT), thrombin time (TT), prothrombin time (PT), and fibrinogen (FIB).

Chest tube management followed standardized institutional criteria throughout the study period. Chest tubes were removed when all of the following conditions were met: (1) absence of air leak, defined as no bubbling during coughing; (2) drainage volume <200 mL over 24 h; and (3) no abnormalities on routine follow-up chest CT. These criteria were applied consistently across all patients in both HBA and non-HBA groups.

The primary outcome was the effect of intrapleural HBA on postoperative coagulation function. Secondary outcomes included the duration of postoperative hospital stay and the incidence of postoperative complications. The 30-day postoperative mortality was also recorded.

Postoperative complications were assessed using predefined institutional criteria. Prolonged air leak was defined as an air leak persisting for more than 5 postoperative days, consistent with commonly used thoracic surgical definitions (17). Lung infection was diagnosed based on new or progressive postoperative pulmonary infiltrates on imaging, together with compatible clinical findings such as fever, leukocytosis or leukopenia, purulent sputum, or the need for antibiotic treatment; microbiological findings were considered supportive when available (18).

### Statistical analysis

To minimize confounding and selection bias arising from the non-randomized assignment of HBA treatment, we employed Inverse Probability of Treatment Weighting (IPTW). The propensity model included clinically relevant baseline covariates: sex, age, BMI, smoking history, comorbidities, surgical approach, TNM stage, resection site and type, lymph node retrieval and stations explored, surgical duration, intraoperative bleeding, pathological types, imaging description, preoperative D-Dimer, INR, APTT, TT, PT, and FIB. Covariate balance after IPTW was assessed using standardized mean differences (SMD), with an SMD < 0.1 considered indicative of good balance.

Continuous variables were presented as mean ± standard deviation if normally distributed, or median and interquartile range (IQR) if non-normally distributed, and were compared using Student’s *t*-test or the Wilcoxon rank-sum test, respectively. Categorical variables were expressed as counts and percentages and compared using the Chi-square test or Fisher’s exact test, as appropriate.

Associations with postoperative complications were examined using binary logistic regression. Predictors of postoperative hospital stay were identified using multivariable linear regression. For both multivariable models, variables with a *p* < 0.05 in univariate analyses were considered candidates. The final models were constructed using a backward elimination procedure with a retention threshold of *p* < 0.05. All statistical tests were two-sided, and a *p*-value < 0.05 was considered statistically significant. Analyses were performed using R software (version 4.1.3; R Foundation for Statistical Computing, Vienna, Austria).

Relationship to prior accepted work. A prior, smaller analysis from our group was based on an earlier subset of the same institutional cohort (approximately *n* = 120 after 1:2 propensity score matching) (8). The present study substantially extends that work by (i) expanding the cohort to *n* = 442 with increased outcome events; (ii) adopting an inverse probability of treatment weighting (IPTW) framework to estimate population-average effects with improved covariate balance; and (iii) broadening endpoints and prespecified subgroup and interaction analyses aligned with thoracic ERAS pathways. The earlier report is cited accordingly, and we have not reused any text, tables, or figures from that publication.

Cohort overlap statement. The two studies partially overlap in calendar time and institution; however, the current analysis includes additional eligible patients accrued thereafter and re-estimates effects under a distinct causal framework. All analyses and visualizations are newly generated.

## Results

### Demographic and baseline characteristics

**Figure 1:**
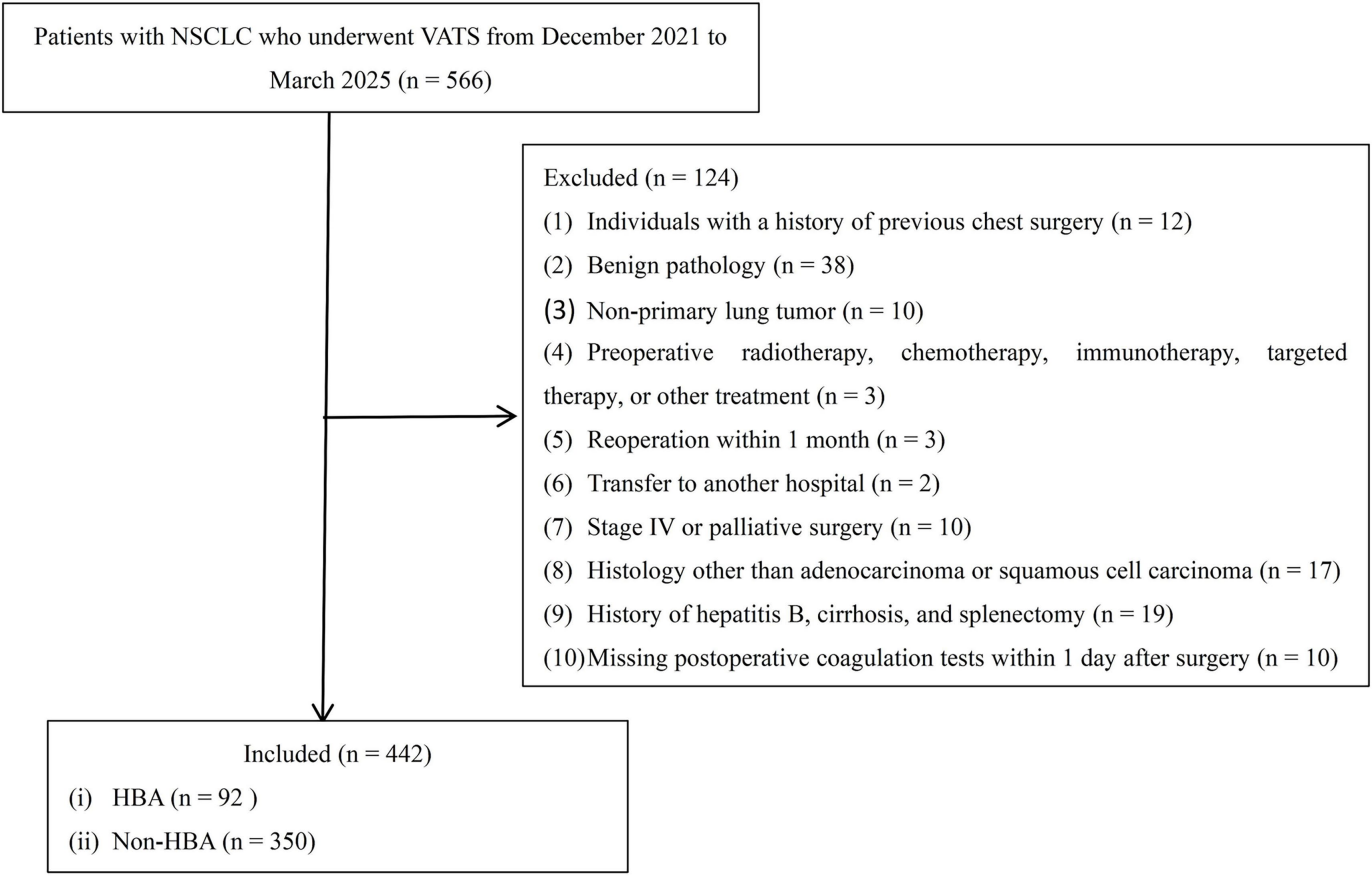
Flow diagram showing the schema of study selection for patients with lung cancer patients. APTT, activated partial thromboplastin time; BMI, body mass index; CI, confidence interval; FIB, fibrinogen; HBA, hemocoagulase Bothrops atrox; IPTW, inverse probability of treatment weighting; INR, international normalized ratio; M-VATS, multiportal video-assisted thoracoscopic surgery; PT, prothrombin time; TT, thrombin time; TNM stage, tumor, node, and metastasis stage; U-VATS, uniportal video-assisted thoracoscopic surgery; VATS, video-assisted thoracoscopic surgery.

The patient selection process is detailed in Figure 1. From an initial cohort of 566 patients, 442 with stage IA NSCLC who underwent VATS were included in the final analysis, comprising 92 in the HBA group and 350 in the non-HBA group. The overall cohort had a mean age of 62.7 ± 10.7 years, included 204 males (46.1%) and 238 females (53.9%), and was distributed across TNM stages as follows: 34.6% IA1 (*n* = 153), 41.4% IA2 (*n* = 183), and 24.0% IA3 (*n* = 106). Uniportal VATS (U-VATS) was performed in 286 patients (64.7%) and multiportal VATS (M-VATS) in 156 (35.3%).

As presented in Table 1, several baseline characteristics were imbalanced between the two groups before adjustment. For instance, the HBA group had a higher proportion of patients undergoing U-VATS (89.1% vs. 58.3%) and wedge resections (60.9% vs. 19.7%). To address these and other potential confounding factors, Inverse Probability of Treatment Weighting (IPTW) was applied. After IPTW, excellent balance was achieved across baseline covariates—including demographics, comorbidities, tumor characteristics, and preoperative laboratory values—with all standardized mean differences (SMDs) below 0.1.

**Table 1:** Baseline demographic and clinicopathological characteristics before and after IPTW.

Variables	Before IPTW					After IPTW				
	Total (*n* = 442)	HBA (*n* = 92)	Non-HBA (*n* = 350)	*p*	SMD	Total (−)	HBA (−)	Non-HBA (−)	*p*	SMD
Sex, *n* (%)				0.193	0.153				0.043	0.049
Male	204 (46.1)	48 (52.2)	156 (44.6)			381 (49.4)	180 (53.6)	201 (46.2)		
Female	238 (53.9)	44 (47.8)	194 (55.4)			390 (50.6)	156 (46.4)	234 (53.8)		
Smoking, *n* (%)	131 (29.6)	27 (29.4)	104 (29.7)	0.945	0.008	246 (31.9)	113 (33.6)	133 (30.6)	0.378	0.064
Comorbidities, *n* (%)	174 (39.4)	29 (31.5)	145 (41.3)	0.083	0.207	301 (39.0)	126 (37.4)	175 (40.2)	0.422	0.057
Age (Mean ± SD)	62.7 ± 10.7	61.6 ± 11.9	63.0 ± 10.3	0.264	0.126	63.1 ± 11.3	63.1 ± 12.1	63.1 ± 10.7	0.984	0.001
BMI (Mean ± SD)	22.9 ± 3.5	22.8 ± 3.5	22.9 ± 3.5	0.920	0.012	23.0 ± 3.7	23.2 ± 4.0	22.9 ± 3.5	0.172	0.090
Pathological types, *n* (%)				0.845	0.023				0.997	<0.001
Adenocarcinoma	387 (87.6)	80 (87.0)	307 (87.7)			676 (87.8)	295 (87.8)	381 (87.8)		
Squamous cell carcinoma	55 (12.4)	12 (13.0)	43 (12.3)			94 (12.2)	41 (12.2)	53 (12.2)		
TNM stage, *n* (%)				0.075	0.359				<0.001	0.093
IA1	153 (34.6)	34 (37.0)	119 (34.0)			288 (37.3)	123 (36.5)	165 (37.9)		
IA2	183 (41.4)	46 (50.0)	137 (39.1)			327 (42.4)	168 (49.9)	159 (36.6)		
IA3	106 (24.0)	12 (13.0)	94 (26.9)			157 (20.3)	46 (13.6)	111 (25.5)		
Imaging description, *n* (%)				0.558	0.211				0.089	0.037
Ground glass nodule	113 (25.6)	29 (31.5)	84 (24.0)			200 (25.9)	98 (29.1)	102 (23.5)		
Mixed nodule	150 (33.9)	25 (27.2)	125 (35.7)			260 (33.7)	101 (30.0)	159 (36.6)		
Solid nodule	179 (40.5)	38 (41.3)	141 (40.3)			311 (40.3)	138 (40.9)	173 (39.9)		

BMI, body mass index; CI, confidence interval; SMD, standardized mean difference.

Following this adjustment, comparative analyses of the weighted cohort revealed distinct outcome profiles. Compared to the non-HBA group, patients who received HBA exhibited a statistically significant prolongation of prothrombin time (PT) (12.83 ± 0.99 s vs. 12.31 ± 1.50 s, *p* < 0.001) and a reduction in fibrinogen (FIB) levels (322.06 ± 96.68 mg/dL vs. 353.52 ± 122.65 mg/dL, *p* < 0.001). In contrast, activated partial thromboplastin time (APTT), thrombin time (TT), and D-Dimer levels were comparable between the groups.

Clinically, HBA administration was associated with significantly higher postoperative albumin (ALB), a lower incidence of postoperative complications, and a shorter postoperative hospital stay (Table 2).

**Table 2:** Surgical characteristics, perioperative outcomes, and laboratory parameters before and after IPTW.

Variables	Before IPTW					After IPTW				
	Total (*n* = 442)	HBA (*n* = 92)	Non-HBA (*n* = 350)	*p*	SMD	Total (−)	HBA (−)	Non-HBA (−)	*p*	SMD
Surgical approach, *n* (%)				<0.001	0.748				<0.001	0.077
U-VATS	286 (64.7)	82 (89.1)	204 (58.3)			555 (72.1)	276 (82.1)	279 (64.3)		
M-VATS	156 (35.3)	10 (10.9)	146 (41.7)			215 (27.9)	60 (17.9)	155 (35.7)		
Resection site, *n* (%)				0.861	0.200				<0.001	0.039
Right upper	138 (31.2)	31 (33.7)	107 (30.6)			248 (32.1)	113 (33.4)	135 (31.1)		
Right middle	29 (6.6)	6 (6.5)	23 (6.6)			46 (6.0)	20 (5.9)	26 (6.0)		
Right lower	83 (18.8)	12 (13.0)	71 (20.3)			127 (16.5)	34 (10.1)	93 (21.4)		
Left upper	111 (25.1)	24 (26.1)	87 (24.9)			206 (26.7)	103 (30.5)	103 (23.7)		
Left lower	81 (18.3)	19 (20.7)	62 (17.7)			145 (18.8)	68 (20.1)	77 (17.7)		
Type of lung resection, *n* (%)				<0.001	0.945				<0.001	0.051
Lobectomy	233 (52.7)	23 (25.0)	210 (60.0)			348 (45.2)	118 (35.1)	230 (53.0)		
Segmental	84 (19.0)	13 (14.1)	71 (20.3)			172 (22.3)	86 (25.6)	86 (19.8)		
Wedge	125 (28.3)	56 (60.9)	69 (19.7)			250 (32.5)	132 (39.3)	118 (27.2)		
Intraoperative bleeding volume [M(P25, P75)]	50 (50, 100)	50 (18, 50)	100 (50, 100)	<0.001	0.746	50 (50, 100)	50 (20, 100)	50 (50, 100)	<0.001	0.050
Surgical duration [M(P25, P75)]	120 (86, 160)	80 (59, 110)	130 (95, 173)	<0.001	0.646	110 (82, 153)	106 (80, 140)	120 (90, 165)	<0.001	0.078
Number of mediastinal lymph nodes retrieved [M(P25, P75)]	3 (0, 9)	3 (1, 5)	4 (0, 10)	0.158	0.131	3 (0, 9)	2 (1, 7)	3 (0, 9)	0.130	0.081
Mediastinal lymph node stations explored [M(P25, P75)]	2 (0, 3)	2 (1, 3)	2 (0, 3)	0.321	0.000	1 (0, 3)	1 (1, 3)	2 (0, 3)	0.334	0.041
Drainage volume [M(P25, P75)]	700 (378, 1,174)	400 (200, 623)	800 (466, 1,250)	<0.001	0.635	650 (300, 1,051)	470 (210, 770)	775 (426, 1,200)	<0.001	0.635*
Drainage time [M(P25, P75)]	4 (3, 8)	3 (2, 4)	5 (3, 9)	<0.001	0.447	4 (3, 6)	5 (3, 9)	5 (3, 9)	<0.001	0.447*
Postoperative complications, *n* (%)	33 (7.4)	5 (5.4)	28 (8.0)	0.143	0.100	74 (9.6)	22 (6.5)	52 (12.0)	<0.001	0.100*
Postoperative hospital stay [M(P25, P75)]	7.5 (5.4, 11.4)	4.4 (3.4, 6.0)	8.5 (5.6, 12.0)	<0.001	0.828	6.0 (4.0, 9.0)	5.0 (4.0, 6.0)	8.0 (5.0, 12.0)	<0.001	0.828*
Preoperative ALB (Mean ± SD)	43.30 ± 4.41	43.54 ± 3.94	43.24 ± 4.53	0.520	0.073	43.07 ± 4.18	42.93 ± 3.74	43.18 ± 4.50	0.398	0.060
Preoperative D-Dimer [M(P25, P75)]	0.28 (0.17, 0.47)	0.29 (0.20, 0.36)	0.28 (0.16, 0.53)	0.997	0.035	0.30 (0.18, 0.44)	0.32 (0.23, 0.43)	0.27 (0.15, 0.48)	<0.001	0.098
Preoperative INR (Mean ± SD)	0.97 (0.91, 1.03)	1.03 ± 0.13	0.96 ± 0.09	<0.001	0.697	0.98 ± 0.09	1.00 ± 0.09	0.96 ± 0.09	<0.001	0.065
Preoperative APTT (Mean ± SD)	28.04 ± 3.71	29.91 ± 3.24	28.04 ± 3.71	<0.001	0.685	28.44 ± 3.45	29.11 ± 3.11	27.92 ± 3.61	<0.001	0.053
Preoperative TT (Mean ± SD)	17.59 ± 2.13	16.83 ± 2.44	17.79 ± 2.00	0.001	0.429	17.58 ± 2.39	17.51 ± 2.85	17.63 ± 1.97	0.516	0.050
Preoperative PT (Mean ± SD)	11.46 ± 1.12	12.06 ± 1.41	11.30 ± 0.98	<0.001	0.630	11.56 ± 1.00	11.74 ± 1.00	11.41 ± 0.98	<0.001	0.031
Preoperative FIB (Mean ± SD)	287.03 ± 77.39	299.93 ± 70.40	283.64 ± 78.87	0.072	0.219	289.44 ± 73.28	289.93 ± 61.12	289.08 ± 81.54	<0.001	<0.001
Postoperative ALB (Mean ± SD)	34.28 ± 4.00	35.72 ± 2.94	33.90 ± 4.16	<0.001	0.508	34.77 ± 3.75	35.50 ± 3.13	34.20 ± 4.09	<0.001	0.508*
Postoperative D-Dimer [M(P25, P75)]	1.33 (0.78, 2.50)	1.19 (0.75, 2.04)	1.41 (0.78, 2.97)	0.102	0.288	1.41 (0.76, 3.19)	1.32 (0.76, 3.38)	1.41 (0.78, 2.59)	0.832	0.288*
Postoperative INR [M(P25, P75)]	1.04 (0.96, 1.12)	1.11 (1.05, 1.17)	1.03 (0.94, 1.11)	<0.001	0.179	1.09 ± 0.34	1.11 ± 0.09	1.08 ± 0.44	0.28	0.179*
Postoperative APTT (Mean ± SD)	30.73 ± 4.64	32.68 ± 3.90	30.21 ± 4.69	<0.001	0.573	31.17 ± 4.26	31.95 ± 3.41	31.57 ± 4.74	<0.001	0.573*
Postoperative TT (Mean ± SD)	16.30 ± 1.95	15.97 ± 2.20	16.38 ± 1.88	0.103	0.202	16.23 ± 2.08	16.22 ± 2.33	16.24 ± 1.84	0883	0.202*
Postoperative PT (Mean ± SD)	12.35 ± 1.47	13.06 ± 1.15	12.16 ± 1.49	<0.001	0.675	12.54 ± 1.33	12.83 ± 0.99	12.31 ± 1.50	<0.001	0.675*
Postoperative FIB (Mean ± SD)	343.20 ± 115.07	348.05 ± 120.06	324.73 ± 91.96	0.185	0.219	339.79 ± 113.07	322.06 ± 96.68	353.52 ± 122.65	<0.001	0.219*

APTT, activated partial thromboplastin time; CI, confidence interval; FIB, fibrinogen; HBA, hemocoagulase Bothrops atrox; IPTW, inverse probability of treatment weighting; INR, international normalized ratio; M(P25, P75), median (25th percentile,75th percentile); M-VATS, multiportal video-assisted thoracoscopic surgery; PT, prothrombin time; SE, standard error; TT, thrombin time; TNM stage, Tumor, Node, and Metastasis stage; U-VATS, uniportal video-assisted thoracoscopic surgery; VATS, video-assisted thoracoscopic surgery.

^*^For postoperative outcomes, SMD values are reported as effect size measures and are not used to assess IPTW covariate balance.

### Primary outcomes

#### Postoperative coagulation profiles

The impact of intrapleural HBA on coagulation parameters was first examined using linear regression (Table 3). Multivariable models were then constructed to adjust for prespecified confounders.

**Table 3:** Univariate and multivariate analysis results of intrapleural HBA and postoperative coagulation parameters.

Parameter	Univariable linear regression analyses					Multivariable linear regression analyses				
	*β*	S. E	Beta	*p*	95% CI	*β*	S. E	Beta	*p*	95% CI
ALB	1.31	0.27	0.17	<0.001	0.78, 1.83	0.65	0.23	0.09	0.004	0.21, 1.10
D-Dimer	−0.68	0.39	−0.06	0.079	−1.43, 0.08	-				
INR	0.02	0.03	0.04	0.337	−0.03, 0.07	-				
APTT	1.38	0.31	0.16	<0.001	0.78, 1.98	0.47	0.28	0.05	0.100	−0.09, 1.02
TT	−0.02	0.15	−0.01	0.879	−0.32, 0.27	-				
PT	0.53	0.09	0.20	<0.001	0.34, 0.71	0.28	0.09	0.10	0.001	0.11, 0.45
FIB	−31.46	8.14	−0.14	<0.001	−47.44, −15.49	−33.33	7.71	−0.15	<0.001	−48.46, −18.20

In univariate analyses, HBA exposure was associated with higher serum ALB (*β* = 1.31, 95% CI 0.78–1.83, *p* < 0.001), a modest but statistically significant prolongation of PT (*β* = 0.53, 95% CI 0.34–0.71, *p* < 0.001), and a reduction in FIB (*β* = −31.46, 95% CI -47.44 to −15.49, *p* < 0.001). APTT was also slightly prolonged (*β* = 1.38, 95% CI 0.78–1.98, *p* < 0.001), whereas TT, INR and D-Dimer did not differ between groups (*p* > 0.05).

After multivariable adjustment, HBA remained an independent predictor of higher ALB (*β* = 0.65, 95% CI 0.21–1.10, *p* = 0.004), longer PT (*β* = 0.28, 95% CI 0.11–0.45, *p* = 0.001) and lower FIB (*β* = −33.33, 95% CI -48.46 to −18.20, *p* < 0.001); the APTT effect was attenuated and no longer significant (*β* = 0.470, 95% CI -0.09 to 1.02, *p* = 0.100).

Detailed univariable and multivariable results for each coagulation parameter are presented in Supplementary Tables 1–7.

### Secondary endpoints

#### Postoperative complications

A total of 33 postoperative complications occurred, with 5 in the HBA group and 28 in the non-HBA group. In the IPTW-weighted cohort, the incidence of postoperative complications remained lower in the HBA group than in the non-HBA group (6.5% vs. 12.0%; *p* < 0.001; Table 2). The major postoperative complications included lung infections in 28 patients, prolonged air leaks in 18 patients, poor wound healing in 3 patients, and postoperative delirium in 4 patients. There were no deaths during hospitalization or within 30 days after discharge, and no patients experienced postoperative bleeding or thrombosis.

Multivariable logistic regression revealed that HBA exposure emerged as a protective factor, reducing complication odds by 65% (odds ratio [OR] = 0.35, 95% CI: 0.19–0.63, *p* = 0.001). Conversely, advanced age (OR = 1.05, *p* = 0.004), higher TNM stage (IA3) (OR = 2.17, *p* = 0.007), and M-VATS surgical approach (OR = 4.23, *p* < 0.001) increased complication risks. Resection type also played a role: segmental (OR = 0.35, *p* = 0.014) and wedge resections (OR = 0.44, *p* = 0.032) exhibited lower complication rates compared to lobectomy. Intraoperative factors, such as mediastinal lymph node stations explored (OR = 1.61, *p* < 0.001), were associated with higher complications, while preoperative ALB levels (OR = 0.93, *p* = 0.017) demonstrated a protective effect (Table 4).

**Table 4:** Results of univariate and multivariable binary logistic regression analyses for postoperative complications.

Variables	Univariate logistic regression analyses					Multivariable logistic regression analyses				
	*β*	S. E	Wald	*p*	95% CI for Exp (B)	*β*	S. E	Wald	*p*	95% CI for Exp (B)
HBA	−1.34	0.25	28.53	<0.001	0.26 (0.16, 0.43)	−1.06	0.31	11.97	0.001	0.35 (0.19, 0.63)
Sex										
Male	Refer									
Female	−0.34	0.21	2.74	0.098	0.71 (0.48, 1.07)					
Smoking	0.48	0.21	5.27	0.022	1.62 (1.07, 2.43)	0.23	0.27	0.71	0.398	1.25 (0.74, 2.14)
Comorbidities	0.56	0.21	7.49	0.006	1.75 (1.17, 2.61)	0.28	0.27	1.02	0.313	1.32 (0.77, 2.25)
Age	0.05	0.01	22.66	<0.001	1.05 (1.03, 1.08)	0.05	0.02	8.07	0.004	1.05 (1.01, 1.08)
BMI	−0.11	0.03	13.45	<0.001	0.89 (0.84, 0.95)	−0.10	0.04	6.19	0.013	0.91 (0.84, 0.98)
Pathological types										
Adenocarcinoma	Refer									
Squamous cell carcinoma	0.38	0.28	1.84	0.175	1.47 (0.84, 2.56)					
TNM stage										
IA1	Refer					Refer				
IA2	0.46	0.26	3.29	0.070	1.59 (0.96, 2.63)	-				
IA3	1.16	0.27	18.14	<0.001	3.20 (1.87, 5.45)	0.78	0.29	7.387	0.007	2.17 (1.24, 3.79)
Surgical approach										
U-VATS	Refer					Refer				
M-VATS	1.54	0.21	52.86	<0.001	4.67 (3.08, 7.07)	1.44	0.29	24.21	<0.001	4.23 (2.38, 7.52)
Imaging description										
Ground glass nodule	Refer					Refer				
Mixed nodule	0.80	0.33	5.67	0.017	2.21 (1.15, 4.26)	0.04	0.40	0.01	0.912	1.05 (0.48, 2.29)
Solid nodule	1.27	0.31	16.18	<0.001	3.54 (1.91, 6.56)	−0.12	0.40	0.09	0.759	0.88 (0.40, 1.95)
Resection site										
Right upper	Refer					Refer				
Right middle	−2.23	1.02	4.74	0.030	0.11 (0.02, 0.80)	−2.93	1.09	7.21	0.007	0.05 (0.01, 0.45)
Right lower	−0.51	0.33	2.35	0.125	0.60 (0.32, 1.15)					
Left upper	0.17	0.24	0.50	0.480	1.19 (0.74, 1.92)					
Left lower	−0.51	0.32	2.60	0.107	0.60 (0.31, 1.12)					
Type of lung resection										
Lobectomy	Refer					Refer				
Segmental	−1.58	0.34	21.45	<0.001	0.21 (0.11, 0.40)	−1.06	0.43	6.02	0.014	0.35 (0.15, 0.81)
Wedge	−1.38	0.27	25.79	<0.001	0.25 (0.15, 0.43)	−0.83	0.39	4.60	0.032	0.44 (0.21, 0.93)
Intraoperative bleeding volume	0.00	0.00	16.34	<0.001	1.00 (1.00, 1.01)	0.00	0.00	0.81	0.367	1.00 (0.99, 1.00)
Surgical duration	0.01	0.00	11.92	0.001	1.01 (1.00, 1.01)	−0.01	0.00	2.94	0.086	1.00 (0.99, 1.00)
Number of mediastinal lymph nodes retrieved	0.11	0.02	35.81	<0.001	1.12 (1.08, 1.16)	−0.08	0.04	4.28	0.039	0.92 (0.85, 1.00)
Mediastinal lymph node stations explored	0.44	0.06	53.70	<0.001	1.55 (1.38, 1.74)	0.478	0.105	20.83	<0.001	1.61 (1.31, 1.98)
Preoperative ALB	−0.09	0.02	14.26	<0.001	0.91 (0.87, 0.96)	−0.076	0.032	5.72	0.017	0.93 (0.87, 0.99)
Preoperative D-Dimer	0.24	0.11	4.73	0.030	1.27 (1.02, 1.57)	0.048	0.126	0.15	0.702	1.05 (0.82, 1.34)
Preoperative INR	−1.51	1.17	1.65	0.199	0.22 (0.22, 2.21)					
Preoperative APTT	−0.08	0.03	6.53	0.011	0.93 (0.87, 0.98)	0.017	0.04	0.21	0.645	1.02 (0.95, 1.09)
Preoperative TT	0.10	0.04	5.66	0.017	1.10 (1.02, 1.19)	−0.062	0.057	1.19	0.275	0.94 (0.84, 1.05)
Preoperative PT	−0.12	0.11	1.24	0.266	0.89 (0.72, 1.10)					
Preoperative FIB	0.00	0.00	0.19	0.665	1.00 (1.00, 1.00)					

#### Postoperative hospital stay

Regarding postoperative hospital stay, multivariable linear regression highlighted HBA exposure as a key determinant, shortening hospital stay by 2.06 days (95% CI: −2.61 to −1.50, *p* < 0.001). Surgical approach again influenced recovery duration, with M-VATS prolonging stay by 1.9 days (95% CI: 1.17–2.55, *p* < 0.001) compared to U-VATS. Resection type and site were critical, as segmental (*β* = −2.40 days, *p* < 0.001) and wedge resections (*β* = −1.98, *p* < 0.001) reduced stay, while right middle lobe resection shortened it by 3.08 days (95% CI: −4.25 to −1.92, *p* < 0.001). Laboratory markers also contributed, with postoperative ALB (*β* = −0.11, *p* = 0.011) inversely associated with stay duration (Table 5).

**Table 5:** Results of univariable and multivariable linear regression analyses for postoperative hospital stay.

Variables	Univariate linear regression analyses					Multivariable linear regression analyses				
	*β*	S. E	Beta	*p*	95% CI	*β*	S. E	Beta	*p*	95% CI
HBA	−3.34	0.33	−0.35	<0.001	−3.98, −2.71	−2.06	0.28	−0.21	<0.001	−2.61, −1.50
Sex										
Male	Refer									
Female	−0.56	0.34	−0.06	0.101	−1.24, 0.11					
Smoking	1.26	0.37	0.12	0.001	0.54, 1.98	0.89	0.31	0.09	0.005	0.27, 1.51
Comorbidities	1.34	0.35	0.14	<0.001	0.65, 2.02	0.44	0.31	0.05	0.153	−0.16, 1.04
Age	0.09	0.02	0.22	<0.001	0.07, 0.12	0.04	0.01	0.09	0.010	0.01, 0.07
BMI	−0.10	0.05	−0.07	0.040	−0.19, −0.00	0.01	0.04	0.01	0.741	−0.07, 0.09
Pathological types										
Adenocarcinoma	Refer					Refer				
Squamous cell carcinoma	1.21	0.52	0.08	0.021	0.18, 2.24	−0.77	0.49	−0.05	0.118	−1.73, 0.20
TNM stage										
IA1	Refer					Refer				
IA2	0.46	0.26	3.29	0.070	−0.96, 2.63	-				
IA3	1.16	0.27	18.14	<0.001	1.87, 5.45	1.57	0.34	0.13	<0.001	0.90, 2.25
Surgical approach										
U-VATS	Refer					Refer				
M-VATS	3.99	0.36	0.38	<0.001	3.29, 4.69	1.86	0.35	0.18	<0.001	1.17, 2.55
Imaging description										
Ground glass nodule	Refer					Refer				
Mixed nodule	1.31	0.44	0.13	<0.001	0.44, 2.18	−0.25	0.37	−0.03	0.496	−0.98, 0.47
Solid nodule	2.11	0.43	0.22	<0.001	1.27, 2.94	−0.6	0.40	−0.06	0.141	−1.39, 0.20
Resection site										
Right upper	Refer					Refer				
Right middle	−2.51	0.76	−0.13	0.001	−4.01, −1.02	−3.08	0.59	−0.15	<0.001	−4.25, −1.92
Right lower	−0.45	0.52	−0.04	0.384	−1.47, 0.57					
Left upper	−0.38	0.45	−0.04	0.395	−1.26, 0.50					
Left lower	−0.95	0.5	−0.08	0.057	−1.93, 0.03					
Type of lung resection										
Lobectomy	Refer					Refer				
Segmental	−3.68	0.40	−0.32	<0.001	−4.47, −2.88	−2.4	0.42	−0.21	<0.001	−3.28, −1.62
Wedge	−4.27	0.36	−0.42	<0.001	−4.98, −3.57	−1.98	0.41	−0.19	<0.001	−2.77, −1.18
Intraoperative bleeding volume	0.01	0.00	0.29	<0.001	0.01, 0.02	0.00	0.00	0.07	0.039	0.00, 0.01
Surgical duration	0.03	0.00	0.33	<0.001	0.02, 0.04	0.01	0.00	0.05	0.165	−0.00, 0.01
Number of mediastinal lymph nodes retrieved	0.33	0.03	0.35	<0.001	0.27, 0.40	−0.10	0.05	−0.11	0.042	−0.20, −0.00
Mediastinal lymph node stations explored	1.00	0.09	0.37	<0.001	0.82, 1.18	0.62	0.13	0.23	<0.001	0.37, 0.87
Preoperative ALB	−0.20	0.04	−0.18	<0.001	−0.28, −0.12	−0.04	0.04	−0.04	0.262	−0.12, 0.03
Preoperative D-Dimer	0.68	0.24	0.10	0.004	0.22, 1.15	0.09	0.19	0.01	0.645	−0.28, 0.46
Preoperative INR*	−8.64	1.84	−0.17	<0.001	−12.25, −5.02					
Preoperative APTT	−0.26	0.05	−0.19	<0.001	−0.35, −0.16	−0.05	0.05	−0.03	0.317	−0.13, 0.04
Preoperative TT	0.43	0.07	0.22	<0.001	0.29, 0.57	0.24	0.07	0.12	0.001	0.10, 0.38
Preoperative PT*	−0.84	0.17	−0.18	<0.001	−1.17, −0.50					
Preoperative FIB	0.00	0.00	−0.05	0.150	−0.01, 0.00					
Postoperative ALB	−0.43	0.04	−0.34	<0.001	−0.51, −0.35	−0.11	0.05	−0.09	0.011	−0.20, −0.03
Postoperative D-Dimer	0.09	0.03	0.10	0.008	0.02, 0.15	0.03	0.03	0.04	0.22	−0.02, 0.08
Postoperative INR	−1.29	0.51	−0.09	0.011	−2.29, −0.29	0.17	0.45	0.01	0.715	−0.72, 1.05
Postoperative APTT	−0.15	0.04	−0.14	<0.001	−0.23, −0.07	−0.06	0.04	−0.05	0.177	−0.14, 0.03
Postoperative TT	0.31	0.08	0.14	<0.001	0.15, 0.47	−0.03	0.08	−0.02	0.674	−0.19, 0.12
Postoperative PT	−0.74	0.13	−0.21	<0.001	−0.99, −0.49	0.12	0.14	0.03	0.404	−0.16, 0.40
Postoperative FIB	0.00	0.00	0.03	0.489	−0.00, 0.00					

*Collinearity.

## Discussion

This study shows that a single intrapleural injection of hemocoagulase Bothrops atrox (HBA) is independently associated with a distinct postoperative coagulation profile and with clinically meaningful benefits in patients with stage IA NSCLC undergoing VATS. After multivariable adjustment, HBA exposure was linked to a modest prolongation of prothrombin time (PT; *β* = 0.28 s) and a reduction in plasma fibrinogen (FIB; *β* = −33.33 mg/dL), whereas activated partial thromboplastin time (APTT) and thrombin time (TT) showed no independent change (APTT: *p* = 0.100; TT: *p* = 0.879). These laboratory changes co-occurred with superior early outcomes—namely, 65% lower odds of postoperative complications (OR = 0.35) and a 2.06-day shorter postoperative length of stay (LOS)—without an observed increase in thromboembolic events or 30-day mortality, supporting the safety of a single 10-unit intrapleural dose (16, 19).

The pre-IPTW disparities in surgical approach and resection type likely reflect underlying clinical practice patterns rather than chance. At our institution, intrapleural HBA tended to be used more frequently in less invasive procedures, such as U-VATS and wedge resections. This may relate to the presence of exposed pleural or parenchymal surfaces prone to minor oozing, where topical agents can be readily applied to reduce postoperative drainage. In contrast, in more extensive operations such as lobectomy, hemostasis is typically achieved through established vascular control and standard surgical techniques, which may reduce the perceived need for adjunctive agents. These decisions, largely driven by surgeon preference and intraoperative judgment, highlight the non-random nature of treatment allocation and further support the use of IPTW to account for baseline differences.

Compared with our earlier matched analysis, the present IPTW study provides population-average causal estimates in a larger cohort, confirms the postoperative coagulation signature *in vivo*, and links that signature more directly to chest-tube–centered recovery milestones within ERAS pathways.

Mechanistically, this selective signature is coherent with HBA’s thrombin-like serine protease activity. HBA preferentially cleaves fibrinogen at Aα-chains to release fibrinopeptide A, promoting localized fibrin generation at the pleural injury site (10, 11). The observed fall in FIB is thus consistent with substrate consumption *in situ*, while the stability of APTT and TT underscores pathway selectivity (i.e., no broad activation of the intrinsic or common pathway) (20). In parallel, contemporary translational work that targets discrete nodes of the hemostatic system—such as inhibition of α2-antiplasmin—demonstrates the feasibility of achieving hemostatic efficacy without incurring excess systemic bleeding, offering convergent external plausibility for a targeted approach (21, 22). Taken together, these lines of evidence support a model in which intrapleural HBA induces localized fibrin formation and limited systemic footprint, reconciling the coagulation pattern we observed with the clinical safety profile.

Building on this mechanistic interpretation, the clinical signal in our cohort is consistent with balanced, localized hemostasis. Despite a reduction in FIB, no patient crossed the commonly cited threshold of 1.5 g/L associated with heightened bleeding risk in peri-trauma and operative settings (19). Clinically, HBA was independently associated with fewer complications and shorter hospitalization, with no recorded postoperative thrombosis or bleeding. These observations align with the concept of balanced hemostasis—effective local clot formation without systemic coagulopathy—and argue for the biological plausibility of intrapleural HBA as an adjunct within VATS pathways.

Importantly, safety considerations appear to depend on route and exposure. Reports of hypofibrinogenemia are concentrated in contexts of repeated intravenous use, particularly beyond 5 days, where the incidence of severe FIB depletion has ranged from 6.5 to 50% in historical cohorts (15, 23). By contrast, our single-dose intrapleural approach was not associated with bleeding or thrombotic penalties, underscoring the roles of delivery compartment and dosing frequency in modulating risk.

Beyond the direct hemostatic effect, the magnitude of clinical improvement we observed—a 65% reduction in overall complications and a 2.06-day reduction in LOS—suggests a broader pathway toward enhanced recovery. By lowering postoperative drainage volume and shortening drainage duration, intrapleural HBA likely reduces hematoma/seroma formation, removes a substrate for bacterial colonization, and improves tissue apposition, thereby mitigating risks of pulmonary infection and prolonged air leak (PAL). This view is concordant with thoracic surgical evidence in which PAL (commonly defined as >5 days) is a driver of pneumonia and delayed discharge, and with programs showing that strategies reducing drainage duration are associated with lower infectious morbidity (16, 24). The pattern of complications in our cohort is consistent with this pathway.

Consistently, the abbreviated hospital stay serves as a downstream indicator of these upstream effects within modern perioperative pathways, where chest-tube removal functions as a dominant, modifiable milestone. By reducing drainage and facilitating earlier tube removal, HBA directly affects this pivotal event, thereby improving patient comfort, facilitating mobilization, and easing nursing workload. This effect is quantified in contemporary ERAS implementations, which report that each additional day of chest-tube drainage is associated with approximately 0.8 extra hospital days (*β* ≈ 0.8; 95% CI 0.5–1.1) (25). The concordance between these external data and our results underscores HBA’s role as an effective upstream lever on discharge-relevant outcomes. Notably, this capacity to accelerate discharge is not restricted to thoracic surgery; it finds parallels in other surgical disciplines, such as urology, where HBA administration similarly shortened hospital stay (14), reinforcing a consistent benefit across operative contexts.

Although pain was not directly measured, a plausible adjunctive pathway links intrapleural fluid burden to inflammation and symptom intensity. Residual pleural blood can perpetuate visceral irritation and a pro-inflammatory state, potentially exacerbating pain and increasing opioid requirements. Contemporary studies demonstrate that early postoperative inflammatory markers (e.g., plasma interleukin-6) scale with surgical stress and correlate with complications and delayed recovery (26), supporting the premise that limiting pleural fluid may attenuate this inflammatory cascade.

Together, these mechanistic and clinical strands provide a coherent framework for how localized hemostasis can translate into accelerated recovery, and they set the stage for comparison with the broader literature on surgical applications of hemocoagulase. The hemostatic efficacy and clinical benefits observed in our study resonate with findings across surgical disciplines, reinforcing the broader applicability of hemocoagulase. In a multicenter, phase III, double-blind randomized trial of abdominal incision hemostasis, Wei et al. showed rapid local hemostasis with low wound-level bleeding (mean hemostatic time 36.8 ± 18.7 s; hemorrhagic volume 3.77 ± 3.93 g in the Agkistrodon group) without safety concerns; global intraoperative blood loss and length of stay were not assessed (27). In a prospective, single-blinded randomized trial of geriatric hip hemiarthroplasty (*n* = 96), Qiu et al. reported significant reductions in intra- and postoperative blood loss, total drainage, mean transfusion volume, and transfusion rates (all *p* < 0.05) without increased adverse events (28, 29). These external data—spanning local surgical hemostasis and a high-bleeding-risk orthopedic model—are directionally consistent with our observations of reduced drainage burden and accelerated recovery following intrapleural HBA.

Within this context, our investigation extends prior work by providing one of the most granular, clinically anchored characterizations of postoperative coagulation changes after intrapleural HBA in VATS, moving beyond bleeding endpoints to a laboratory signature that aligns with its pharmacology. In addition, we linked this signature to recovery-relevant outcomes—a 65% reduction in overall complications and a 2.06-day shorter length of stay—together with concomitant reductions in drainage volume and duration. While prior studies primarily documented clinical bleeding endpoints (14, 27, 28), our analysis offers in-vivo support for enzymatic fibrinogen consumption at the pleural injury site. Specifically, the distinct coagulation pattern we identified—a decrease in fibrinogen (*β* = −33.33 mg/dL, *p* < 0.001) coupled with a prolongation of PT (*β* = 0.28 s, *p* = 0.001)—is mechanistically coherent with thrombin-like serine proteases and their high Aα-chain specificity (10, 11, 30–33).

Furthermore, our study expands the evaluation of HBA beyond hemostasis to encompass robust recovery endpoints. The 65% reduction in overall complications and the 2.06-day shortening of postoperative LOS suggest that HBA functions not merely as a hemostatic adjunct but as a contributor to enhanced recovery pathways in thoracic surgery. The significant reductions in postoperative drainage volume and duration provide a plausible physiological bridge between localized hemostasis and these superior clinical outcomes, consistent with established surgical principles in which prolonged air leak and sustained drainage are key determinants of morbidity and delayed discharge (24, 34). Application of IPTW to balance a comprehensive set of baseline covariates further strengthens internal validity relative to many previous observational cohorts, yielding a more reliable estimate of the treatment association with intrapleural HBA. Taken together, these data support a model in which targeted, localized fibrin generation translates into a lower drainage burden and fewer complications while preserving intrinsic/common-pathway stability, thereby advancing recovery in the VATS setting for early-stage lung cancer and motivating subsequent analyses of indications, dosing, and generalizability.

The present findings have several direct implications for thoracic surgical practice. First, they support the selective use of a single 10-unit intrapleural HBA injection as a pragmatic adjunct that aligns with Enhanced Recovery After Surgery (ERAS) objectives in stage IA NSCLC undergoing VATS. By shortening drainage duration and lowering drainage volume—two modifiable levers within thoracic ERAS pathways—HBA exposure was associated with earlier chest-tube removal and mobilization, translating into a materially shorter postoperative length of stay without an observable safety penalty (35). These associations are directionally concordant with contemporary ERAS evidence in lung resection, in which optimization of chest-tube strategy and expedited tube removal have been shown to reduce morbidity and shorten hospitalization in high-quality cohorts and programs (25, 36).

From a decision-making standpoint, our data provide a quantitative basis for targeted HBA use in patients whose perioperative profile makes drainage-related delay likely—for example, more extensive resections or borderline preoperative hemostatic reserve—while acknowledging the need for prospective validation. Recent multicenter randomized work has shown that early chest-tube removal irrespective of drainage volume is non-inferior to conventional thresholds after anatomic pulmonary resection (36); additionally, a separate multicenter trial supports physiology-based, weight-adjusted removal thresholds without compromising safety, further aligning chest-tube management with risk-guided practice (37). In parallel, implementation studies of thoracic ERAS have linked protocolized postoperative elements—including early tube removal, early ambulation, and opioid-sparing analgesia—to significant reductions in morbidity and length of stay on a propensity-balanced basis (38). In combination with our results, these data suggest a coherent, actionable pathway: minimizing pleural burden facilitates earlier tube removal, which accelerates functional milestones and discharge (38).

A further implication concerns postoperative laboratory interpretation in patients receiving intrapleural HBA. The phenotype of isolated PT prolongation with reduced fibrinogen that we observed is best interpreted as the expected pharmacological footprint of localized fibrin generation/consumption at the pleural injury site—rather than a generalized coagulopathy requiring corrective products—when uncoupled from bleeding signs. This view is mechanistically anchored in contemporary coagulation biology and venom-enzyme specificity work, which emphasize the Aα-chain selectivity of thrombin-like proteases and its predictable impact on fibrin-formation kinetics (11, 30–32). In practice, recognizing this pattern may prevent over-correction (e.g., empiric plasma or fibrinogen concentrate) in otherwise stable patients and encourages integration of clinical context with laboratory data.

This study has several key strengths that bolster the reliability of its findings. Firstly, the use of Inverse Probability of Treatment Weighting (IPTW) successfully balanced extensive baseline differences between the groups, particularly in surgical approach and resection extent, thereby substantially reducing selection bias and strengthening the causal interpretation of the associations. Secondly, the analysis leveraged a comprehensive and detailed dataset encompassing demographics, surgical specifics, and complete pre- and postoperative laboratory profiles, which allowed for robust multivariable adjustment and a granular examination of outcomes. Finally, by focusing exclusively on a homogeneous cohort of patients with pathological stage IA NSCLC, the study enhances its internal validity and provides clear insights applicable to this specific surgical population.

Several limitations of this study warrant consideration. Firstly, its single-center, retrospective nature, despite the application of IPTW, cannot entirely exclude residual confounding from unmeasured variables. Secondly, the lack of a standardized protocol for HBA administration and the absence of blinding may have introduced performance and detection bias in outcome assessment. These limitations highlight the need for future prospective, randomized controlled trials to validate our findings, optimize dosing strategies, evaluate cost-effectiveness, and explore potential long-term impacts on oncological outcomes.

In conclusion, intrapleural HBA injection in stage IA NSCLC patients undergoing VATS significantly impacts postoperative coagulation function while demonstrating clear clinical benefits. This intervention is independently associated with reduced complications and shorter hospital stays, supporting its role in enhancing recovery after thoracic surgery. Further prospective studies are needed to validate these findings and establish optimal implementation protocols.

## Data Availability

The raw data supporting the conclusions of this article will be made available by the authors, without undue reservation.

## Glossary

ALB: Albumin
APTT: Activated partial thromboplastin time
BMI: Body mass index
CI: Confidence interval
ERAS: Enhanced recovery after surgery
FIB: Fibrinogen
HBA: Hemocoagulase Bothrops atrox
IQR: Interquartile range
IPTW: Inverse probability of treatment weighting
IASLC: International Association for the Study of Lung Cancer
INR: International normalized ratio
LOS: Length of stay
M(P25, P75): Median (25th percentile, 75th percentile)
M-VATS: Multiportal video-assisted thoracoscopic surgery
NSCLC: Non–small cell lung cancer
OR: Odds ratio
PAL: Prolonged air leak
PT: Prothrombin time
SMD: Standardized mean difference
SE: Standard error
TT: Thrombin time
TNM stage: Tumor, node and metastasis stage
U-VATS: Uniportal video-assisted thoracoscopic surgery
VATS: Video-assisted thoracoscopic surgery

## References

[1] Rami-Porta R Nishimura KK Giroux DJ Detterbeck F Cardillo G Edwards JG . The International Association for the Study of Lung Cancer lung Cancer staging project: proposals for revision of the TNM stage groups in the forthcoming (ninth) edition of the TNM classification for lung cancer. J Thorac Oncol. (2024) 19:1007–27. doi: 10.1016/j.jtho.2024.02.011, 38447919

[2] Riely GJ Wood DE Ettinger DS Aisner DL Akerley W Bauman JR . Non-small cell lung Cancer, version 4.2024, NCCN clinical practice guidelines in oncology. J Natl Compr Cancer Netw. (2024) 22:249–74. doi: 10.6004/jnccn.2204.0023, 38754467

[3] Harris RJ Law JJ Hao L Situ D Dittberner FA Bendixen M . (2026) Survival outcome of VATS compared with open lobectomy for lung cancer: an individual patient data meta-analysis of randomised trials. Lancet. (2026) 407:1182–1190. doi: 10.1016/S0140-6736(26)00031-0 41864749

[4] Piwkowski C Gabryel P Campisi A Orłowski TM Zieliński M Rzyman W . Ninety-day mortality of thoracoscopic vs open lobectomy: a large multicenter cohort study. Ann Thorac Surg. (2023) 115:693–9. doi: 10.1016/j.athoracsur.2022.07.050, 35988738

[5] Pan JM Watkins AA Stock CT Moffatt-Bruce SD Servais EL. The surgical renaissance: advancements in video-assisted thoracoscopic surgery and robotic-assisted thoracic surgery and their impact on patient outcomes. Cancers. (2024) 16:3086. doi: 10.3390/cancers16173086, 39272946 PMC11393871

[6] Yao L Wang W. Effect of intraoperative blood loss on postoperative pulmonary complications in patients undergoing video-assisted thoracoscopic surgery. Turk Gogus Kalp Damar Cerrahisi Derg. (2021) 29:347–53. doi: 10.5606/tgkdc.dergisi.2021.20657, 34589253 PMC8462118

[7] Chen D Chen Y Wang M. Analysis of hemostatic effect and safety of local spray treatment with hemocoagulase Bothrops atrox for injection after resection of colon polyps. Altern Ther Health Med. (2023) 29:406–11. 37632968

[8] Xie Y Han J Yang J Xiong W Ye J Yang A . Intrathoracic hemocoagulase Bothrops atrox injection efficacy in thoracoscopic lung cancer surgery. Medicine (Baltimore). (2025) 104:e46001. doi: 10.1097/MD.0000000000046001, 41239649 PMC12622591

[9] Goldstraw P Chansky K Crowley J Rami-Porta R Asamura H Eberhardt WE . The IASLC lung Cancer staging project: proposals for revision of the TNM stage groupings in the forthcoming (eighth) edition of the TNM classification for lung Cancer. J Thorac Oncol. (2016) 11:39–51. doi: 10.1016/j.jtho.2015.09.009, 26762738

[10] Oliveira AL Viegas MF da Silva SL Soares AM Ramos MJ Fernandes PA. The chemistry of snake venom and its medicinal potential. Nat Rev Chem. (2022) 6:451–69. doi: 10.1038/s41570-022-00393-7, 35702592 PMC9185726

[11] Wolberg AS. Fibrinogen and fibrin: synthesis, structure, and function in health and disease. J Thromb Haemost. (2023) 21:3005–15. doi: 10.1016/j.jtha.2023.08.014, 37625698 PMC10592048

[12] Feller T Connell SDA Ariёns RAS. Why fibrin biomechanical properties matter for hemostasis and thrombosis. J Thromb Haemost. (2022) 20:6–16. doi: 10.1111/jth.15531, 34528378

[13] Risman RA Sen M Tutwiler V Hudson NE. Deconstructing fibrin(ogen) structure. J Thromb Haemost. (2025) 23:368–80. doi: 10.1016/j.jtha.2024.10.024, 39536819 PMC11786978

[14] Torigoe K Yamashita A Abe S Muta K Mukae H Nishino T. Effect of hemocoagulase on the prevention of bleeding after percutaneous renal biopsy. Toxins. (2022) 14:223. doi: 10.3390/toxins14030223, 35324720 PMC8951486

[15] Ma W Zhao T Yu L Liu W Wang H Zhao P. Incidence, clinical features, and risk factors of hemocoagulase-induced hypofibrinogenemia: a retrospective real-world study. Medicine (Baltimore). (2024) 103:e37773. doi: 10.1097/MD.0000000000037773, 38608074 PMC11018171

[16] Lan D Jiao B Song S Wang M Zhang X Huang X . Effects of batroxobin on the antithrombotic system in patients with cerebral venous thrombosis: clues to mechanisms. CNS Neurosci Ther. (2024) 30:e14861. doi: 10.1111/cns.14861, 39097912 PMC11298196

[17] Kent MS Mitzman B Diaz-Gutierrez I Khullar OV Fernando HC Backhus L . The Society of Thoracic Surgeons expert consensus document on the Management of Pleural Drains after Pulmonary Lobectomy: expert consensus document. Ann Thorac Surg. (2024) 118:764–77. doi: 10.1016/j.athoracsur.2024.04.016, 38723882

[18] Imperatori A Nardecchia E Dominioni L Sambucci D Spampatti S Feliciotti G . Surgical site infections after lung resection: a prospective study of risk factors in 1,091 consecutive patients. J Thorac Dis. (2017) 9:3222–31. doi: 10.21037/jtd.2017.08.122, 29221299 PMC5708450

[19] Moore EE Moore HB Kornblith LZ Neal MD Hoffman M Mutch NJ . Trauma-induced coagulopathy. Nat Rev Dis Primers. (2021) 7:30. doi: 10.1038/s41572-021-00264-3, 33927200 PMC9107773

[20] Wolberg AS Sang Y. Fibrinogen and factor XIII in venous thrombosis and thrombus stability. Arterioscler Thromb Vasc Biol. (2022) 42:931–41. doi: 10.1161/ATVBAHA.122.317164, 35652333 PMC9339521

[21] Singh S Kumar P Padwad YS Jaffer FA Reed GL. Targeting fibrinolytic inhibition for venous thromboembolism treatment: overview of an emerging therapeutic approach. Circulation. (2024) 150:884–98. doi: 10.1161/CIRCULATIONAHA.124.069728, 39250537 PMC11433585

[22] Lan D Jiao B Song S Wang M Zhang X Huang X . Effects of batroxobin on the antithrombotic system in patients with cerebral venous thrombosis: clues to mechanisms. CNS Neurosci Ther. (2024) 30:e14861. doi: 10.1111/cns.14861, 39097912 PMC11298196

[23] Ma W Zhao T Yu L Liu W Wang H Zhao P. Incidence, clinical features, and risk factors of hemocoagulase-induced hypofibrinogenemia: a retrospective real-world study. Medicine. (2024) 103:e37773. doi: 10.1097/MD.0000000000037773, 38608074 PMC11018171

[24] Chopra A Hu K Judson MA Fabian T Nabagiez JP Feustel PJ . Association between drainage-dependent prolonged air leak after partial lung resection and clinical outcomes: a prospective cohort study. Ann Am Thorac Soc. (2022) 19:389–98. doi: 10.1513/AnnalsATS.202103-235OC, 34715010

[25] Dyas AR Stuart CM Bronsert MR Kelleher AD Bata KE Cumbler EU . Anatomic lung resection outcomes after implementation of a universal thoracic ERAS protocol across a diverse health care system. Ann Surg. (2024) 279:1062–9. doi: 10.1097/SLA.0000000000006243, 38385282 PMC11087203

[26] Neff TA Braun J Rana D Puhan M Filipovic M Seeberger M . Interleukin-6 is an early plasma marker of severe postoperative complications in thoracic surgery: exploratory results from a substudy of a randomized controlled multicenter trial. Anesth Analg. (2022) 134:123–32. doi: 10.1213/ANE.0000000000005639, 34132704

[27] Wei JM Zhu MW Zhang ZT Jia ZG He XD Wan YL . A multicenter, phase III trial of hemocoagulase Agkistrodon: hemostasis, coagulation, and safety in patients undergoing abdominal surgery. Chin Med J. (2010) 123:589–93. 20367987

[28] Qiu M Zhang X Cai H Xu Z Lin H. The impact of hemocoagulase for improvement of coagulation and reduction of bleeding in fracture-related hip hemiarthroplasty geriatric patients: a prospective, single-blinded, randomized, controlled study. Injury. (2017) 48:914–9. doi: 10.1016/j.injury.2016.11.028, 28238301

[29] Torigoe K Yamashita A Abe S Muta K Mukae H Nishino T. Effect of hemocoagulase on the prevention of bleeding after percutaneous renal biopsy. Toxins. (2022) 14:223. doi: 10.3390/toxins14030223, 35324720 PMC8951486

[30] Veizaj D den Exter PL Bos MHA. Russell's viper venom: from diagnostic to bypassing agent for hemophilia? J Thromb Haemost. (2023) 21:1429–31. doi: 10.1016/j.jtha.2023.02.026, 37179074

[31] Nellenbach K Kyu A Guzzetta N Brown AC. Differential sialic acid content in adult and neonatal fibrinogen mediates differences in clot polymerization dynamics. Blood Adv. (2021) 5:5202–14. doi: 10.1182/bloodadvances.2021004417, 34555851 PMC9153052

[32] Moore GW. Snake venoms in diagnostic hemostasis and thrombosis. Semin Thromb Hemost. (2022) 48:145–60. doi: 10.1055/s-0041-1732465, 34384127

[33] Nellenbach K Kyu A Guzzetta N Brown AC. Differential sialic acid content in adult and neonatal fibrinogen mediates differences in clot polymerization dynamics. Blood Adv. (2021) 5:5202–14. doi: 10.1182/bloodadvances.2021004417, 34555851 PMC9153052

[34] Wolberg AS Sang Y. Fibrinogen and factor XIII in venous thrombosis and thrombus stability. Arterioscler Thromb Vasc Biol. (2022) 42:931–41. doi: 10.1161/ATVBAHA.122.317164, 35652333 PMC9339521

[35] Dyas AR Stuart CM Bronsert MR Kelleher AD Bata KE Cumbler EU . Anatomic lung resection outcomes after implementation of a universal thoracic ERAS protocol across a diverse health care system. Ann Surg. (2024) 279:1062–9. doi: 10.1097/SLA.0000000000006243, 38385282 PMC11087203

[36] Takamochi K Haruki T Oh S Endo M Funai K Kitamura Y . Early chest tube removal regardless of drainage volume after anatomic pulmonary resection: a multicenter, randomized, controlled trial. J Thorac Cardiovasc Surg. (2024) 168:401–410.e1. doi: 10.1016/j.jtcvs.2023.10.050, 38348845

[37] Gioutsos K Ehrenreich L Azenha LF Quapp CS Kocher GJ Lutz JA . Randomized controlled trial of thresholds for drain removal after anatomic lung resection. Ann Thorac Surg. (2024) 117:1103–9. doi: 10.1016/j.athoracsur.2023.09.011, 37734641

[38] Haro GJ Sheu B Marcus SG Sarin A Campbell L Jablons DM . Perioperative lung resection outcomes after implementation of a multidisciplinary, evidence-based thoracic ERAS program. Ann Surg. (2021) 274:e1008–13. doi: 10.1097/SLA.0000000000003719, 31851005

