# Supplementary material for: Intrapleural hemocoagulase Bothrops atrox and early outcomes after VATS for stage IA non-small cell lung cancer
Source: Front Med (Lausanne). 2026 Apr 10;13:1774067. doi: 10.3389/fmed.2026.1774067 (PMC13106133; doi:10.3389/fmed.2026.1774067)
Supplement: Supplementary file 1 [file Table_1.DOCX]

| Supplementary Table 1. Results of univariate and multivariable linear regression analyses for postoperative ALB | | | | | | | | | | |
| --- | --- | --- | --- | --- | --- | --- | --- | --- | --- | --- |
| Variables | Univariable linear regression analyses | | | | | Multivariable linear regression analyses | | | | |
|  | β | S.E | Beta | P | 95% CI | β | S.E | Beta | P | 95% CI |
| HBA | 1.31 | 0.27 | 0.17 | <0.001 | 0.78, 1.83 | 0.65 | 0.23 | 0.09 | 0.004 | 0.21, 1.10 |
| Sex |  |  |  |  |  |  |  |  |  |  |
| Male | Refer |  |  |  |  |  |  |  |  |  |
| Female | 0.29 | 0.27 | 0.04 | 0.280 | -0.24, 0.82 |  |  |  |  |  |
| Smoking | -0.08 | 0.29 | -0.01 | 0.771 | -0.65, 0.49 |  |  |  |  |  |
| Comorbidities | -1.08 | 0.28 | -0.14 | <0.001 | -1.62, -0.54 | -0.07 | 0.24 | -0.01 | 0.772 | -0.55, 0.41 |
| Age | -0.11 | 0.01 | -0.32 | <0.001 | -0.13, -0.09 | -0.03 | 0.01 | -0.08 | 0.038 | -0.05, 0.00 |
| BMI | 0.09 | 0.04 | 0.09 | 0.018 | 0.02, 0.16 | 0.10 | 0.03 | 0.10 | 0.002 | 0.04, 0.17 |
| Pathological types |  |  |  |  |  |  |  |  |  |  |
| Adenocarcinoma | Refer |  |  |  |  | Refer |  |  |  |  |
| Squamous cell carcinoma | -2.85 | 0.40 | -0.25 | <0.001 | -3.63, -2.06 | -1.32 | 0.38 | -0.12 | 0.001 | -2.08, -0.57 |
| TNM stage |  |  |  |  |  |  |  |  |  |  |
| ⅠA1 | Refer |  |  |  |  | Refer |  |  |  |  |
| ⅠA2 | -0.39 | 0.34 | -0.05 | 0.247 | -1.06, 0.27 | - |  |  |  |  |
| ⅠA3 | -0.84 | 0.42 | -0.08 | 0.042 | -1.66, -0.03 | -0.38 | 0.28 | -0.04 | 0.179 | -0.93, 0.17 |
| Surgical approach |  |  |  |  |  |  |  |  |  |  |
| U-VATS | Refer |  |  |  |  | Refer |  |  |  |  |
| M-VATS | -1.82 | 0.29 | -0.22 | <0.001 | -2.40, -1.24 | -0.55 | 0.27 | -0.07 | 0.046 | -1.09, -0.01 |
| Imaging Description |  |  |  |  |  |  |  |  |  |  |
| Ground glass nodule | Refer |  |  |  |  | Refer |  |  |  |  |
| Mixed nodule | -1.37 | 0.38 | -0.16 | <0.001 | -2.12, -0.62 | 0.59 | 0.30 | 0.07 | 0.051 | -0.00, 1.18 |
| Solid nodule | -2.64 | 0.37 | -0.31 | <0.001 | -3.36, -1.92 | 0.80 | 0.32 | 0.10 | 0.013 | 0.17, 1.43 |
| Resection Site |  |  |  |  |  |  |  |  |  |  |
| Right upper | Refer |  |  |  |  | Refer |  |  |  |  |
| Right middle | -1.83 | 0.67 | -0.10 | 0.006 | -3.14, -0.53 | 0.49 | 0.49 | 0.03 | 0.318 | -0.47, 1.45 |
| Right lower | -0.43 | 0.45 | -0.04 | 0.340 | -1.32, 0.46 | - |  |  |  |  |
| Left upper | -1.1 | 0.39 | -0.12 | 0.005 | -1.87, -0.33 | 0.46 | 0.27 | 0.05 | 0.084 | -0.06, 0.98 |
| Left lower | -1.09 | 0.44 | -0.10 | 0.012 | -1.95, -0.24 | -0.09 | 0.30 | -0.01 | 0.763 | -0.69, 0.50 |
| Type of lung resection |  |  |  |  |  |  |  |  |  |  |
| Lobectomy | Refer |  |  |  |  |  |  |  |  |  |
| Segmental | 0.36 | 0.39 | 0.04 | 0.357 | -0.41, 1.12 |  |  |  |  |  |
| Wedge | 0.59 | 0.35 | 0.07 | 0.091 | -0.09, 1.27 |  |  |  |  |  |
| Intraoperative bleeding volume | -0.01 | 0.00 | -0.41 | <0.001 | -0.02, -0.01 | -0.01 | 0.00 | -0.22 | <0.001 | -0.01, -0.01 |
| Surgical duration | -0.03 | 0.00 | -0.39 | <0.001 | -0.03, -0.02 | -0.01 | 0.00 | -0.19 | <0.001 | -0.02, -0.01 |
| Number of mediastinal lymph nodes retrieved | -0.15 | 0.03 | -0.20 | <0.001 | -0.20, -0.10 | 0.03 | 0.04 | 0.03 | 0.651 | -0.05, 0.10 |
| Mediastinal lymph node stations explored | -0.40 | 0.08 | -0.19 | <0.001 | -0.55, -0.25 | -0.15 | 0.10 | -0.07 | 0.143 | -0.35, 0.05 |
| Preoperative ALB | 0.35 | 0.03 | 0.39 | <0.001 | 0.29, 0.41 | 0.22 | 0.03 | 0.25 | <0.001 | 0.17, 0.28 |
| Preoperative D-Dimer | -0.60 | 0.19 | -0.12 | 0.001 | -0.96, -0.23 | 0.04 | 0.15 | 0.01 | 0.262 | -0.26, 0.34 |
| Preoperative INR | 1.93 | 1.46 | 0.05 | 0.187 | -0.94, 4.81 |  |  |  |  |  |
| Preoperative APTT | 0.19 | 0.04 | 0.17 | <0.001 | 0.11, 0.26 | 0.11 | 0.03 | 0.11 | 0.001 | 0.05, 0.18 |
| Preoperative TT | -0.17 | 0.06 | -0.11 | 0.003 | -0.28, -0.06 | -0.09 | 0.05 | -0.06 | 0.107 | -0.20, 0.02 |
| Preoperative PT | 0.25 | 0.13 | 0.07 | 0.066 | -0.02, 0.51 |  |  |  |  |  |
| Preoperative FIB | -0.01 | 0.00 | -0.17 | <0.001 | -0.01, -0.02 | -0.01 | 0.00 | -0.16 | <0.001 | -0.01, -0.01 |
| APTT, activated partial thromboplastin time; BMI, body mass index; CI, confidence interval; FIB, fibrinogen; HBA, hemocoagulase bothrops atrox; IPTW, inverse probability of treatment weighting; INR, international normalized ratio; M(P25,P75), median(25th percentile,75th percentile); M-VATS, multiportal video-assisted thoracoscopic surgery; PT, prothrombin time; SE, standard error; TT, thrombin time; TNM stage, Tumor, Node, and Metastasis stage; U-VATS, uniportal video-assisted thoracoscopic surgery; VATS, video-assisted thoracoscopic surgery. | | | | | | | | | | |
