# Supplementary material for: Intrapleural hemocoagulase Bothrops atrox and early outcomes after VATS for stage IA non-small cell lung cancer
Source: Front Med (Lausanne). 2026 Apr 10;13:1774067. doi: 10.3389/fmed.2026.1774067 (PMC13106133; doi:10.3389/fmed.2026.1774067)
Supplement: Supplementary file 2 [file Table_2.DOCX]

| Supplementary Table 2. Results of univariate and multivariable linear regression analyses for postoperative D-Dimer | | | | | | | | | | |
| --- | --- | --- | --- | --- | --- | --- | --- | --- | --- | --- |
| Variables | Univariable linear regression analyses | | | | | Multivariable linear regression analyses | | | | |
|  | β | S.E | Beta | P | 95% CI | β | S.E | Beta | P | 95% CI |
| HBA | -0.68 | 0.39 | -0.06 | 0.079 | -1.43, 0.08 |  |  |  |  |  |
| Sex |  |  |  |  |  |  |  |  |  |  |
| Male | Refer |  |  |  |  |  |  |  |  |  |
| Female | 0.29 | 0.27 | 0.04 | 0.280 | -0.24, 0.82 |  |  |  |  |  |
| Smoking | -0.08 | 0.29 | -0.01 | 0.771 | -0.65, 0.49 |  |  |  |  |  |
| Comorbidities |  |  |  |  |  |  |  |  |  |  |
| Age | 0.08 | 0.02 | 0.18 | <0.001 | 0.05, 0.12 | 0.06 | 0.02 | 0.13 | 0.002 | 0.02, 0.10 |
| BMI | -0.05 | 0.05 | -0.03 | 0.381 | -0.15, 0.06 |  |  |  |  |  |
| Pathological types |  |  |  |  |  |  |  |  |  |  |
| Adenocarcinoma | Refer |  |  |  |  |  |  |  |  |  |
| Squamous cell carcinoma | 0.31 | 0.59 | 0.02 | 0.594 | -0.84, 1.46 |  |  |  |  |  |
| TNM stage |  |  |  |  |  |  |  |  |  |  |
| ⅠA1 | Refer |  |  |  |  |  |  |  |  |  |
| ⅠA2 | 0.8 | 0.43 | 0.07 | 0.064 | -0.05, 1.64 |  |  |  |  |  |
| ⅠA3 | 0.08 | 0.53 | 0.01 | 0.876 | -0.95, 1.12 |  |  |  |  |  |
| Surgical approach |  |  |  |  |  |  |  |  |  |  |
| U-VATS | Refer |  |  |  |  |  |  |  |  |  |
| M-VATS | -0.05 | 0.43 | -0.01 | 0.908 | -0.89, 0.79 |  |  |  |  |  |
| Imaging Description |  |  |  |  |  |  |  |  |  |  |
| Ground glass nodule | Refer |  |  |  |  | Refer |  |  |  |  |
| Mixed nodule | 0.87 | 0.50 | 0.08 | 0.082 | -0.11, 1.85 | - |  |  |  |  |
| Solid nodule | 1.16 | 0.48 | 0.11 | 0.016 | 0.21, 2.10 | 0.17 | 0.43 | 0.02 | 0.696 | -0.67, 1.00 |
| Resection Site |  |  |  |  |  |  |  |  |  |  |
| Right upper | Refer |  |  |  |  | Refer |  |  |  |  |
| Right middle | 1.68 | 0.85 | 0.08 | 0.049 | 0.01, 3.34 | 1.52 | 0.81 | 0.07 | 0.060 | -0.07, 3.10 |
| Right lower | -0.45 | 0.58 | -0.03 | 0.436 | -1.59, 0.69 |  |  |  |  |  |
| Left upper | 0.78 | 0.50 | 0.07 | 0.118 | -0.20, 1.77 |  |  |  |  |  |
| Left lower | -0.32 | 0.56 | -0.02 | 0.567 | -1.41, 0.77 |  |  |  |  |  |
| Type of lung resection |  |  |  |  |  |  |  |  |  |  |
| Lobectomy | Refer |  |  |  |  | Refer |  |  |  |  |
| Segmental | -0.46 | 0.5 | -0.04 | 0.353 | -1.43, 0.51 | - |  |  |  |  |
| Wedge | -0.88 | 0.44 | -0.08 | 0.046 | -1.75, -0.02 | -0.15 | 0.46 | -0.01 | 0.740 | -1.06, 0.75 |
| Intraoperative bleeding volume | 0.01 | 0.00 | 0.10 | 0.007 | 0.00, 0.01 | 0.00 | 0.00 | 0.03 | 0.402 | -0.00, 0.01 |
| Surgical duration | 0.01 | 0.00 | 0.13 | <0.001 | 0.01, 0.02 | 0.01 | 0.00 | 0.07 | 0.107 | -0.00, 0.02 |
| Number of mediastinal lymph nodes retrieved | 0.03 | 0.04 | 0.03 | 0.454 | -0.05, 0.10 | -0.05 | 0.04 | -0.050 | 0.228 | -0.14, 0.03 |
| Mediastinal lymph node stations explored | 0.16 | 0.11 | 0.05 | 0.146 | -0.06, 0.37 |  |  |  |  |  |
| Preoperative ALB | -0.15 | 0.05 | -0.12 | 0.001 | -0.24, -0.06 | -0.03 | 0.05 | -0.02 | 0.551 | -0.13, 0.07 |
| Preoperative D-Dimer | 0.74 | 0.26 | 0.10 | 0.005 | 0.22, 1.26 | 0.52 | 0.266 | 0.071 | 0.052 | -0.01, 1.04 |
| Preoperative INR | 2.29 | 2.08 | 0.04 | 0.270 | -1.79, 6.37 |  |  |  |  |  |
| Preoperative APTT | -0.07 | 0.06 | -0.04 | 0.231 | -0.18, 0.04 |  |  |  |  |  |
| Preoperative TT | 0.04 | 0.08 | 0.02 | 0.590 | -0.11, 0.20 |  |  |  |  |  |
| Preoperative PT | 0.20 | 0.19 | 0.04 | 0.299 | -0.18, 0.57 |  |  |  |  |  |
| Preoperative FIB | 0.00 | 0.00 | 0.02 | 0.592 | 0.00, 0.01 |  |  |  |  |  |
| APTT, activated partial thromboplastin time; BMI, body mass index; CI, confidence interval; FIB, fibrinogen; HBA, hemocoagulase bothrops atrox; IPTW, inverse probability of treatment weighting; INR, international normalized ratio; M(P25,P75), median(25th percentile,75th percentile); M-VATS, multiportal video-assisted thoracoscopic surgery; PT, prothrombin time; SE, standard error; TT, thrombin time; TNM stage, Tumor, Node, and Metastasis stage; U-VATS, uniportal video-assisted thoracoscopic surgery; VATS, video-assisted thoracoscopic surgery. | | | | | | | | | | |
