# Supplementary material for: Intrapleural hemocoagulase Bothrops atrox and early outcomes after VATS for stage IA non-small cell lung cancer
Source: Front Med (Lausanne). 2026 Apr 10;13:1774067. doi: 10.3389/fmed.2026.1774067 (PMC13106133; doi:10.3389/fmed.2026.1774067)
Supplement: Supplementary file 3 [file Table_3.DOCX]

| Supplementary Table 3. Results of univariate and multivariable linear regression analyses for postoperative INR | | | | | | | | | | |
| --- | --- | --- | --- | --- | --- | --- | --- | --- | --- | --- |
| Variables | Univariable linear regression analyses | | | | | Multivariable linear regression analyses | | | | |
|  | β | S.E | Beta | P | 95% CI | β | S.E | Beta | P | 95% CI |
| HBA | 0.02 | 0.03 | 0.04 | 0.337 | -0.03,0.07 |  |  |  |  |  |
| Sex |  |  |  |  |  |  |  |  |  |  |
| Male | Refer |  |  |  |  |  |  |  |  |  |
| Female | -0.03 | 0.02 | -0.04 | 0.269 | -0.08, 0.02 |  |  |  |  |  |
| Smoking | -0.02 | 0.03 | -0.03 | 0.491 | -0.07, 0.03 |  |  |  |  |  |
| Comorbidities | 0.02 | 0.03 | 0.03 | 0.412 | -0.03, 0.07 |  |  |  |  |  |
| Age | 0.00 | 0.00 | 0.03 | 0.355 | -0.00, 0.01 |  |  |  |  |  |
| BMI | 0.00 | 0.00 | 0.04 | 0.293 | -0.00, 0.01 |  |  |  |  |  |
| Pathological types |  |  |  |  |  |  |  |  |  |  |
| Adenocarcinoma | Refer |  |  |  |  |  |  |  |  |  |
| Squamous cell carcinoma | 0.01 | 0.04 | 0.01 | 0.776 | -0.06, 0.08 |  |  |  |  |  |
| TNM stage |  |  |  |  |  |  |  |  |  |  |
| ⅠA1 | Refer |  |  |  |  |  |  |  |  |  |
| ⅠA2 | -0.05 | 0.03 | -0.07 | 0.068 | -0.10, 0.00 |  |  |  |  |  |
| ⅠA3 | -0.05 | 0.03 | -0.06 | 0.113 | -0.12, 0.01 |  |  |  |  |  |
| Surgical approach |  |  |  |  |  |  |  |  |  |  |
| U-VATS | Refer |  |  |  |  | Refer |  |  |  |  |
| M-VATS | -0.13 | 0.03 | -0.17 | <0.001 | -0.18, -0.08 | -0.07 | 0.03 | -0.09 | 0.028 | -0.13, -0.01 |
| Imaging Description |  |  |  |  |  |  |  |  |  |  |
| Ground glass nodule | Refer |  |  |  |  |  |  |  |  |  |
| Mixed nodule | -0.01 | 0.03 | -0.01 | 0.814 | -0.07, 0.06 |  |  |  |  |  |
| Solid nodule | -0.05 | 0.03 | -0.07 | 0.124 | -0.11, 0.01 |  |  |  |  |  |
| Resection Site |  |  |  |  |  |  |  |  |  |  |
| Right upper | Refer |  |  |  |  | Refer |  |  |  |  |
| Right middle | 0.01 | 0.05 | 0.01 | 0.861 | -0.10, 0.12 | - |  |  |  |  |
| Right lower | 0.10 | 0.04 | 0.11 | 0.006 | 0.03, 0.17 | 0.08 | 0.03 | 0.08 | 0.022 | 0.01, 0.14 |
| Left upper | 0.04 | 0.03 | 0.05 | 0.250 | -0.03, 0.10 | - |  |  |  |  |
| Left lower | 0.01 | 0.04 | 0.01 | 0.826 | -0.06, 0.08 | - |  |  |  |  |
| Type of lung resection |  |  |  |  |  |  |  |  |  |  |
| Lobectomy | Refer |  |  |  |  | Refer |  |  |  |  |
| Segmental | 0.03 | 0.03 | 0.04 | 0.299 | -0.03, 0.09 | - |  |  |  |  |
| Wedge | 0.12 | 0.03 | 0.16 | <0.001 | 0.06, 0.17 | 0.06 | 0.03 | 0.08 | 0.048 | 0.00, 0.11 |
| Intraoperative bleeding volume | 0.00 | 0.00 | -0.08 | 0.030 | 0.00, 0.00 | 0.00 | 0.00 | -0.03 | 0.455 | 0.00, 0.00 |
| Surgical duration | 0.00 | 0.00 | -0.06 | 0.104 | -0.00, 0.00 |  |  |  |  |  |
| Number of mediastinal lymph nodes retrieved | -0.01 | 0.00 | -0.12 | 0.001 | -0.01, -0.00 | 0.00 | 0.00 | 0.00 | 0.965 | -0.01, 0.01 |
| Mediastinal lymph node stations explored | -0.02 | 0.01 | -0.10 | 0.005 | -0.03, -0.01 | -0.01 | 0.01 | -0.05 | 0.421 | -0.03, 0.01 |
| Preoperative ALB | 0.00 | 0.00 | 0.03 | 0.471 | 0.00, 0.01 |  |  |  |  |  |
| Preoperative D-Dimer | 0.01 | 0.02 | 0.02 | 0.636 | -0.03, 0.04 |  |  |  |  |  |
| Preoperative INR | 0.55 | 0.13 | 0.15 | <0.001 | 0.29, 0.81 | -0.02 | 0.38 | -0.01 | 0.965 | -0.75, 0.72 |
| Preoperative APTT | 0.01 | 0.00 | 0.13 | 0.001 | 0.01, 0.02 | 0.01 | 0.00 | 0.06 | 0.156 | -0.00, 0.01 |
| Preoperative TT | -0.02 | 0.01 | -0.12 | 0.001 | -0.03, -0.01 | -0.01 | 0.01 | -0.05 | 0.207 | -0.02, 0,00 |
| Preoperative PT | 0.05 | 0.01 | 0.15 | <0.001 | 0.03, 0.08 | 0.03 | 0.03 | 0.07 | 0.463 | -0.04, 0.09 |
| Preoperative FIB | 0.00 | 0.00 | -0.03 | 0.382 | 0.00, 0.00 |  |  |  |  |  |
| APTT, activated partial thromboplastin time; BMI, body mass index; CI, confidence interval; FIB, fibrinogen; HBA, hemocoagulase bothrops atrox; IPTW, inverse probability of treatment weighting; INR, international normalized ratio; M(P25,P75), median(25th percentile,75th percentile); M-VATS, multiportal video-assisted thoracoscopic surgery; PT, prothrombin time; SE, standard error; TT, thrombin time; TNM stage, Tumor, Node, and Metastasis stage; U-VATS, uniportal video-assisted thoracoscopic surgery; VATS, video-assisted thoracoscopic surgery. | | | | | | | | | | |
