# Supplementary material for: Intrapleural hemocoagulase Bothrops atrox and early outcomes after VATS for stage IA non-small cell lung cancer
Source: Front Med (Lausanne). 2026 Apr 10;13:1774067. doi: 10.3389/fmed.2026.1774067 (PMC13106133; doi:10.3389/fmed.2026.1774067)
Supplement: Supplementary file 4 [file Table_4.DOCX]

| Supplementary Table 4. Results of univariate and multivariable linear regression analyses for postoperative APTT | | | | | | | | | | |
| --- | --- | --- | --- | --- | --- | --- | --- | --- | --- | --- |
| Variables | Univariable linear regression analyses | | | | | Multivariable linear regression analyses | | | | |
|  | β | S.E | Beta | P | 95% CI | β | S.E | Beta | P | 95% CI |
| HBA | 1.38 | 0.31 | 0.16 | <0.001 | 0.78, 1.98 | 0.47 | 0.28 | 0.05 | 0.100 | -0.09, 1.02 |
| Sex |  |  |  |  |  |  |  |  |  |  |
| Male | Refer |  |  |  |  |  |  |  |  |  |
| Female | -0.64 | 0.31 | -0.08 | 0.037 | -1.24, -0.04 | -0.29 | 0.38 | -0.03 | 0.442 | -1.03, 0.45 |
| Smoking | 0.65 | 0.33 | 0.07 | 0.049 | 0.00, 1.19 | 0.10 | 0.40 | 0.01 | 0.804 | -0.69, 0.89 |
| Comorbidities | 0.29 | 0.32 | 0.03 | 0.365 | -0.33, 0.90 |  |  |  |  |  |
| Age | 0.02 | 0.01 | 0.07 | 0.072 | -0.00, 0.05 |  |  |  |  |  |
| BMI | -0.08 | 0.04 | -0.07 | 0.054 | -0.16, 0.00 |  |  |  |  |  |
| Pathological types |  |  |  |  |  |  |  |  |  |  |
| Adenocarcinoma | Refer |  |  |  |  |  |  |  |  |  |
| Squamous cell carcinoma | 1.60 | 0.47 | 0.12 | 0.001 | 0.69, 2.52 | 0.59 | 0.48 | 0.05 | 0.22 | -0.35, 1.54 |
| TNM stage |  |  |  |  |  |  |  |  |  |  |
| ⅠA1 | Refer |  |  |  |  |  |  |  |  |  |
| ⅠA2 | 0.07 | 0.35 | 0.01 | 0.838 | -0.61, 0.75 |  |  |  |  |  |
| ⅠA3 | 0.34 | 0.42 | 0.03 | 0.428 | -0.50, 1.17 |  |  |  |  |  |
| Surgical approach |  |  |  |  |  |  |  |  |  |  |
| U-VATS | Refer |  |  |  |  | Refer |  |  |  |  |
| M-VATS | -2.51 | 0.33 | -0.26 | <0.001 | -3.15, -1.86 | -1.28 | 0.35 | -0.14 | <0.001 | -1.97, -0.59 |
| Imaging Description |  |  |  |  |  |  |  |  |  |  |
| Ground glass nodule | Refer |  |  |  |  | Refer |  |  |  |  |
| Mixed nodule | -0.27 | 0.40 | -0.03 | 0.503 | -1.05, 0.52 | - |  |  |  |  |
| Solid nodule | 0.83 | 0.38 | 0.10 | 0.031 | 0.08, 1.59 | 0.74 | 0.32 | 0.09 | 0.019 | 0.12, 1.37 |
| Resection Site |  |  |  |  |  |  |  |  |  |  |
| Right upper | Refer |  |  |  |  |  |  |  |  |  |
| Right middle | 0.09 | 0.68 | 0.01 | 0.893 | -1.25, 1.44 |  |  |  |  |  |
| Right lower | 0.31 | 0.47 | 0.03 | 0.509 | -0.61, 1.22 |  |  |  |  |  |
| Left upper | 0.25 | 0.40 | 0.03 | 0.532 | -0.54, 1.04 |  |  |  |  |  |
| Left lower | 0.14 | 0.45 | 0.01 | 0.757 | -0.74, 1.02 |  |  |  |  |  |
| Type of lung resection |  |  |  |  |  |  |  |  |  |  |
| Lobectomy | Refer |  |  |  |  | Refer |  |  |  |  |
| Segmental | 0.23 | 0.4 | 0.02 | 0.565 | -0.55, 1.10 | - |  |  |  |  |
| Wedge | 0.88 | 0.35 | 0.1 | 0.013 | 0.18, 1.57 | 0.02 | 0.31 | 0.00 | 0.944 | -0.59, 0.64 |
| Intraoperative bleeding volume | 0.00 | 0.00 | -0.08 | 0.020 | -0.01, 0.00 | 0.00 | 0.00 | -0.03 | 0.405 | -0.01, 0.01 |
| Surgical duration | 0.00 | 0.00 | -0.01 | 0.804 | -0.01, 0.01 |  |  |  |  |  |
| Number of mediastinal lymph nodes retrieved | -0.04 | 0.03 | -0.04 | 0.231 | -0.10, 0.02 |  |  |  |  |  |
| Mediastinal lymph node stations explored | 0.05 | 0.09 | 0.02 | 0.566 | -0.12, 0.22 |  |  |  |  |  |
| Preoperative ALB | -0.03 | 0.04 | -0.03 | 0.370 | -0.11, 0.04 |  |  |  |  |  |
| Preoperative D-Dimer | -0.07 | 0.21 | -0.01 | 0.729 | -0.49, 0.34 |  |  |  |  |  |
| Preoperative INR | 17.13 | 1.55 | 0.37 | <0.001 | 14.09, 20.16 | 7.54 | 4.29 | 0.16 | 0.079 | -0.88, 15.97 |
| Preoperative APTT | 0.51 | 0.04 | 0.41 | <0.001 | 0.43, 0.59 | 0.33 | 0.05 | 0.27 | <0.001 | 0.24, 0.42 |
| Preoperative TT | -0.17 | 0.06 | -0.10 | 0.008 | -0.30, -0.04 | -0.06 | 0.06 | -0.03 | 0.367 | -0.18, 0.07 |
| Preoperative PT | 1.51 | 0.14 | 0.36 | <0.001 | 1.23, 1.80 | -0.03 | 0.39 | -0.01 | 0.948 | -0.79, 0.74 |
| Preoperative FIB | 0.00 | 0.00 | -0.03 | 0.419 | -0.01, 0.00 |  |  |  |  |  |
| APTT, activated partial thromboplastin time; BMI, body mass index; CI, confidence interval; FIB, fibrinogen; HBA, hemocoagulase bothrops atrox; IPTW, inverse probability of treatment weighting; INR, international normalized ratio; M(P25,P75), median(25th percentile,75th percentile); M-VATS, multiportal video-assisted thoracoscopic surgery; PT, prothrombin time; SE, standard error; TT, thrombin time; TNM stage, Tumor, Node, and Metastasis stage; U-VATS, uniportal video-assisted thoracoscopic surgery; VATS, video-assisted thoracoscopic surgery. | | | | | | | | | | |
