# Supplementary material for: Intrapleural hemocoagulase Bothrops atrox and early outcomes after VATS for stage IA non-small cell lung cancer
Source: Front Med (Lausanne). 2026 Apr 10;13:1774067. doi: 10.3389/fmed.2026.1774067 (PMC13106133; doi:10.3389/fmed.2026.1774067)
Supplement: Supplementary file 5 [file Table_5.DOCX]

| Supplementary Table 5. Results of univariate and multivariable linear regression analyses for postoperative TT | | | | | | | | | | |
| --- | --- | --- | --- | --- | --- | --- | --- | --- | --- | --- |
| Variables | Univariable linear regression analyses | | | | | Multivariable linear regression analyses | | | | |
|  | β | S.E | Beta | P | 95% CI | β | S.E | Beta | P | 95% CI |
| HBA | -0.02 | 0.15 | -0.01 | 0.879 | -0.32, 0.27 |  |  |  |  |  |
| Sex |  |  |  |  |  |  |  |  |  |  |
| Male | Refer |  |  |  |  | Refer |  |  |  |  |
| Female | 0.45 | 0.15 | 0.11 | 0.003 | 0.16, 0.74 | 0.13 | 0.17 | 0.03 | 0.434 | -0.20, 0.46 |
| Smoking | -0.37 | 0.16 | -0.08 | 0.020 | -0.68, -0.06 | -0.37 | 0.18 | -0.08 | 0.043 | -0.73, -0.01 |
| Comorbidities | -0.11 | 0.15 | -0.03 | 0.456 | -0.41, 0.19 |  |  |  |  |  |
| Age | 0.02 | 0.01 | 0.12 | 0.001 | 0.01, 0.04 | 0.00 | 0.01 | 0.014 | 0.674 | -0.01, 0.02 |
| BMI | 0.02 | 0.01 | 0.12 | 0.001 | 0.01, 0.04 | -0.02 | 0.01 | -0.037 | 0.246 | -0.06, 0.01 |
| Pathological types |  |  |  |  |  |  |  |  |  |  |
| Adenocarcinoma | Refer |  |  |  |  | Refer |  |  |  |  |
| Squamous cell carcinoma | -0.11 | 0.02 | -0.20 | <0.001 | -0.15,-0.07 | 0.47 | 0.21 | 0.07 | 0.024 | 0.06, 0.87 |
| TNM stage |  |  |  |  |  |  |  |  |  |  |
| ⅠA1 | Refer |  |  |  |  | Refer |  |  |  |  |
| ⅠA2 | 0.11 | 0.17 | 0.03 | 0.514 | -0.22, 0.44 | - |  |  |  |  |
| ⅠA3 | 0.54 | 0.21 | 0.11 | 0.009 | 0.14, 0.94 | 0.05 | 0.16 | 0.01 | 0.733 | -0.25, 0.36 |
| Surgical approach |  |  |  |  |  |  |  |  |  |  |
| U-VATS | Refer |  |  |  |  | Refer |  |  |  |  |
| M-VATS | 1.03 | 0.16 | 0.22 | <0.001 | 0.71, 1.35 | 0.40 | 0.16 | 0.09 | 0.010 | 0.10, 0.70 |
| Imaging Description |  |  |  |  |  |  |  |  |  |  |
| Ground glass nodule | Refer |  |  |  |  |  |  |  |  |  |
| Mixed nodule | -0.13 | 0.19 | -0.03 | 0.520 | -0.51, 0.26 |  |  |  |  |  |
| Solid nodule | 0.33 | 0.19 | 0.08 | 0.081 | -0.04, 0.69 |  |  |  |  |  |
| Resection Site |  |  |  |  |  |  |  |  |  |  |
| Right upper | Refer |  |  |  |  | Refer |  |  |  |  |
| Right middle | 0.41 | 0.33 | 0.05 | 0.219 | -0.24, 1.05 | - |  |  |  |  |
| Right lower | -0.24 | 0.23 | -0.04 | 0.288 | -0.68, 0.20 | - |  |  |  |  |
| Left upper | -0.45 | 0.19 | -0.10 | 0.021 | -0.83, -0.07 | -0.15 | 0.19 | -0.03 | 0.414 | -0.52, 0.21 |
| Left lower | -0.41 | 0.22 | -0.08 | 0.056 | -0.84, 0.01 | - |  |  |  |  |
| Type of lung resection |  |  |  |  |  |  |  |  |  |  |
| Lobectomy | Refer |  |  |  |  | Refer |  |  |  |  |
| Segmental | -0.43 | 0.19 | -0.09 | 0.024 | -0.80, -0.06 | 0.11 | 0.19 | 0.02 | 0.578 | -0.27, 0.49 |
| Wedge | -0.99 | 0.17 | -0.22 | <0.001 | -1.32, -0.66 | -0.12 | 0.17 | -0.03 | 0.485 | -0.46, 0.22 |
| Intraoperative bleeding volume | 0.00 | 0.00 | 0.07 | 0.062 | 0.00, 0.00 |  |  |  |  |  |
| Surgical duration | 0.00 | 0.00 | -0.04 | 0.221 | -0.00, 0.00 |  |  |  |  |  |
| Number of mediastinal lymph nodes retrieved | 0.10 | 0.01 | 0.247 | <0.001 | 0.07, 0.13 | 0.06 | 0.02 | 0.14 | 0.010 | 0.01, 0.10 |
| Mediastinal lymph node stations explored | 0.12 | 0.04 | 0.103 | 0.004 | 0.04, 0.20 | -0.14 | 0.06 | -0.12 | 0.013 | -0.26, -0.03 |
| Preoperative ALB | -0.04 | 0.02 | -0.09 | 0.014 | -0.08, -0.01 | 0.00 | 0.02 | 0.00 | 0.915 | -0.03, 0.03 |
| Preoperative D-Dimer | 0.02 | 0.10 | 0.01 | 0.879 | -0.19, 0.22 |  |  |  |  |  |
| Preoperative INR | -4.54 | 0.79 | -0.20 | <0.001 | -6.10, -2.99 | 1.42 | 1.92 | 0.06 | 0.458 | -2.34, 5.19 |
| Preoperative APTT | -0.03 | 0.02 | -0.05 | 0.160 | -0.07, 0.01 |  |  |  |  |  |
| Preoperative TT | 0.49 | 0.03 | 0.56 | <0.001 | 0.43, 0.54 | 0.44 | 0.03 | 0.51 | <0.001 | 0.38, 0.50 |
| Preoperative PT | -0.44 | 0.07 | -0.21 | <0.001 | -0.58, -0.29 | -0.18 | 0.17 | -0.09 | 0.296 | -0.52, 0.16 |
| Preoperative FIB | 0.00 | 0.00 | -0.08 | 0.035 | -0.00, 0.00 | 0.00 | 0.00 | 0.08 | 0.009 | 0.00, 0.00 |
| APTT, activated partial thromboplastin time; BMI, body mass index; CI, confidence interval; FIB, fibrinogen; HBA, hemocoagulase bothrops atrox; IPTW, inverse probability of treatment weighting; INR, international normalized ratio; M(P25,P75), median(25th percentile,75th percentile); M-VATS, multiportal video-assisted thoracoscopic surgery; PT, prothrombin time; SE, standard error; TT, thrombin time; TNM stage, Tumor, Node, and Metastasis stage; U-VATS, uniportal video-assisted thoracoscopic surgery; VATS, video-assisted thoracoscopic surgery. | | | | | | | | | | |
