# Supplementary material for: Intrapleural hemocoagulase Bothrops atrox and early outcomes after VATS for stage IA non-small cell lung cancer
Source: Front Med (Lausanne). 2026 Apr 10;13:1774067. doi: 10.3389/fmed.2026.1774067 (PMC13106133; doi:10.3389/fmed.2026.1774067)
Supplement: Supplementary file 6 [file Table_6.DOCX]

| Supplementary Table 6. Results of univariate and multivariable linear regression analyses for postoperative PT | | | | | | | | | | |
| --- | --- | --- | --- | --- | --- | --- | --- | --- | --- | --- |
| Variables | Univariable linear regression analyses | | | | | Multivariable linear regression analyses | | | | |
|  | β | S.E | Beta | P | 95% CI | β | S.E | Beta | P | 95% CI |
| HBA | 0.53 | 0.09 | 0.2 | <0.001 | 0.34, 0.71 | 0.28 | 0.09 | 0.10 | 0.001 | 0.11, 0.45 |
| Sex |  |  |  |  |  |  |  |  |  |  |
| Male | Refer |  |  |  |  |  |  |  |  |  |
| Female | -0.16 | 0.10 | -0.06 | 0.098 | -0.35, 0.03 |  |  |  |  |  |
| Smoking | 0.07 | 0.10 | 0.02 | 0.517 | -0.14, 0.27 |  |  |  |  |  |
| Comorbidities | 0.00 | 0.10 | 0.00 | 0.977 | -0.20, 0.19 |  |  |  |  |  |
| Age | 0.00 | 0.00 | -0.01 | 0.707 | -0.01, 0.01 |  |  |  |  |  |
| BMI | 0.01 | 0.01 | 0.03 | 0.398 | -0.01, 0.04 |  |  |  |  |  |
| Pathological types |  |  |  |  |  |  |  |  |  |  |
| Adenocarcinoma | Refer |  |  |  |  |  |  |  |  |  |
| Squamous cell carcinoma | 0.04 | 0.15 | 0.01 | 0.791 | -0.25,0.33 |  |  |  |  |  |
| TNM stage |  |  |  |  |  |  |  |  |  |  |
| ⅠA1 | Refer |  |  |  |  | Refer |  |  |  |  |
| ⅠA2 | -0.33 | 0.11 | -0.12 | 0.002 | -0.54, -0.12 | -0.40 | 0.10 | -0.15 | <0.001 | -0.59, -0.22 |
| ⅠA3 | -0.28 | 0.13 | -0.08 | 0.035 | -0.53, -0.02 | -0.10 | 0.12 | -0.03 | 0.368 | -0.33, 0.12 |
| Surgical approach |  |  |  |  |  |  |  |  |  |  |
| U-VATS | Refer |  |  |  |  | Refer |  |  |  |  |
| M-VATS | -1.06 | 0.10 | -0.36 | <0.001 | -1.25, -0.86 | -0.54 | 0.10 | -0.18 | <0.001 | -0.74, -0.34 |
| Imaging Description |  |  |  |  |  |  |  |  |  |  |
| Ground glass nodule | Refer |  |  |  |  |  |  |  |  |  |
| Mixed nodule | -0.10 | 0.13 | -0.04 | 0.420 | -0.35, 0.14 |  |  |  |  |  |
| Solid nodule | -0.09 | 0.12 | -0.03 | 0.472 | -0.32, 0.15 |  |  |  |  |  |
| Resection Site |  |  |  |  |  |  |  |  |  |  |
| Right upper | Refer |  |  |  |  |  |  |  |  |  |
| Right middle | 0.12 | 0.21 | 0.02 | 0.585 | -0.30, 0.53 |  |  |  |  |  |
| Right lower | 0.00 | 0.15 | 0.00 | 0.979 | -0.28, 0.29 |  |  |  |  |  |
| Left upper | 0.10 | 0.13 | 0.03 | 0.417 | -0.14, 0.35 |  |  |  |  |  |
| Left lower | 0.15 | 0.14 | 0.04 | 0.282 | -0.12, 0.42 |  |  |  |  |  |
| Type of lung resection |  |  |  |  |  |  |  |  |  |  |
| Lobectomy | Refer |  |  |  |  | Refer |  |  |  |  |
| Segmental | 0.26 | 0.12 | 0.08 | 0.035 | 0.02, 0.50 | -0.13 | 0.13 | -0.04 | 0.298 | -0.38, 0.12 |
| Wedge | 0.63 | 0.11 | 0.22 | <0.001 | 0.42, 0.84 | 0.06 | 0.12 | 0.02 | 0.603 | -0.17, 0.30 |
| Intraoperative bleeding volume | -0.00 | 0.00 | -0.13 | 0.001 | -0.00, -0.00 | -0.00 | 0.00 | -0.06 | 0.101 | -0.00, 0.00 |
| Surgical duration | -0.00 | 0.00 | -0.09 | 0.014 | -0.00, 0.00 | 0.00 | 0.00 | 0.08 | 0.047 | 0.00, 0.00 |
| Number of mediastinal lymph nodes retrieved | -0.05 | 0.01 | -0.20 | <0.001 | -0.07, -0.04 | -0.03 | 0.02 | -0.11 | 0.041 | -0.06, -0.00 |
| Mediastinal lymph node stations explored | -0.09 | 0.03 | -0.12 | 0.001 | -0.14, -0.04 | 0.01 | 0.04 | 0.01 | 0.836 | -0.07, 0.08 |
| Preoperative ALB | 0.03 | 0.01 | 0.09 | 0.013 | 0.01, 0.05 | 0.02 | 0.01 | 0.07 | 0.041 | 0,00, 0.04 |
| Preoperative D-Dimer | -0.00 | 0.07 | -0.00 | 0.987 | -0.13, 0.13 |  |  |  |  |  |
| Preoperative INR | 6.85 | 0.46 | 0.48 | <0.001 | 5.95, 7.74 | 2.23 | 1.27 | 0.16 | 0.080 | -0.26, 4.72 |
| Preoperative APTT | 0.11 | 0.01 | 0.28 | <0.001 | 0.08, 0.13 | 0.01 | 0.01 | 0.02 | 0.641 | -0.02, 0.03 |
| Preoperative TT | -0.13 | 0.02 | -0.24 | <0.001 | -0.17, -0.09 | -0.02 | 0.02 | -0.03 | 0.402 | -0.05, 0.02 |
| Preoperative PT | 0.63 | 0.04 | 0.48 | <0.001 | 0.55, 0.72 | 0.31 | 0.11 | 0.23 | 0.007 | 0.08, 0.53 |
| Preoperative FIB | 0.00 | 0.00 | -0.00 | 0.974 | -0.00, 0.00 |  |  |  |  |  |
| APTT, activated partial thromboplastin time; BMI, body mass index; CI, confidence interval; FIB, fibrinogen; HBA, hemocoagulase bothrops atrox; IPTW, inverse probability of treatment weighting; INR, international normalized ratio; M(P25,P75), median(25th percentile,75th percentile); M-VATS, multiportal video-assisted thoracoscopic surgery; PT, prothrombin time; SE, standard error; TT, thrombin time; TNM stage, Tumor, Node, and Metastasis stage; U-VATS, uniportal video-assisted thoracoscopic surgery; VATS, video-assisted thoracoscopic surgery. | | | | | | | | | | |
