# Supplementary material for: Intrapleural hemocoagulase Bothrops atrox and early outcomes after VATS for stage IA non-small cell lung cancer
Source: Front Med (Lausanne). 2026 Apr 10;13:1774067. doi: 10.3389/fmed.2026.1774067 (PMC13106133; doi:10.3389/fmed.2026.1774067)
Supplement: Supplementary file 7 [file Table_7.DOCX]

| Supplementary Table 7. Results of univariate and multivariable linear regression analyses for postoperative FIB | | | | | | | | | | |
| --- | --- | --- | --- | --- | --- | --- | --- | --- | --- | --- |
| Variables | Univariable linear regression analyses | | | | | Multivariable linear regression analyses | | | | |
|  | β | S.E | Beta | P | 95% CI | β | S.E | Beta | P | 95% CI |
| HBA | -31.46 | 8.14 | -0.14 | <0.001 | -47.44, -15.49 | -33.33 | 7.71 | -0.15 | <0.001 | -48.46, -18.20 |
| Sex |  |  |  |  |  |  |  |  |  |  |
| Male | Refer |  |  |  |  | Refer |  |  |  |  |
| Female | -16.91 | 8.13 | -0.08 | 0.038 | -32.87, -0.95 | 13.50 | 10.65 | 0.06 | 0.205 | -7.40, 34.40 |
| Smoking | 28.98 | 8.67 | 0.120 | 0.001 | 11.95, 46.00 | 31.35 | 11.13 | 0.13 | 0.005 | 9.50, 53.21 |
| Comorbidities | -7.34 | 8.35 | -0.03 | 0.380 | -23.73, 9.06 |  |  |  |  |  |
| Age | -0.03 | 0.36 | -0.00 | 0.927 | -0.74, 0.67 |  |  |  |  |  |
| BMI | 5.35 | 1.08 | 0.18 | <0.001 | 3.23, 7.47 | 4.42 | 1.12 | 0.15 | <0.001 | 2.23, 6.61 |
| Pathological types |  |  |  |  |  |  |  |  |  |  |
| Adenocarcinoma | Refer |  |  |  |  |  |  |  |  |  |
| Squamous cell carcinoma | -12.32 | 12.41 | -0.04 | 0.321 | -36.69, 12.05 |  |  |  |  |  |
| TNM stage |  |  |  |  |  |  |  |  |  |  |
| ⅠA1 | Refer |  |  |  |  |  |  |  |  |  |
| ⅠA2 | -14.91 | 9.14 | -0.07 | 0.103 | -32.85, 3.03 |  |  |  |  |  |
| ⅠA3 | -15.35 | 11.21 | -0.06 | 0.171 | -37.36, 6.56 |  |  |  |  |  |
| Surgical approach |  |  |  |  |  |  |  |  |  |  |
| U-VATS | Refer |  |  |  |  |  |  |  |  |  |
| M-VATS | 10.77 | 9.08 | 0.04 | 0.236 | -7.05, 28.58 |  |  |  |  |  |
| Imaging Description |  |  |  |  |  |  |  |  |  |  |
| Ground glass nodule | Refer |  |  |  |  | Refer |  |  |  |  |
| Mixed nodule | 32.56 | 10.58 | 0.136 | 0.002 | 11.79, 53.34 | 22.53 | 10.16 | 0.09 | 0.027 | 2.59, 42.48 |
| Solid nodule | 25.02 | 10.19 | 0.109 | 0.014 | 5.00, 45.03 | 18.95 | 10.22 | 0.08 | 0.064 | -1.12, 39.02 |
| Resection Site |  |  |  |  |  |  |  |  |  |  |
| Right upper | Refer |  |  |  |  | Refer |  |  |  |  |
| Right middle | 25.11 | 17.94 | 0.05 | 0.162 | -10.11, 60.33 | - |  |  |  |  |
| Right lower | 8.77 | 12.23 | 0.03 | 0.474 | -15.25, 32.78 | - |  |  |  |  |
| Left upper | 9.31 | 10.58 | 0.04 | 0.379 | -11.46, 30.07 | - |  |  |  |  |
| Left lower | 46.69 | 11.74 | 0.16 | <0.001 | 23.63, 69.74 | 28.61 | 9.91 | 0.1 | 0.004 | 9.14, 48.07 |
| Type of lung resection |  |  |  |  |  |  |  |  |  |  |
| Lobectomy | Refer |  |  |  |  |  |  |  |  |  |
| Segmental | -11.15 | 10.53 | -0.04 | 0.290 | -31.83, 9.53 |  |  |  |  |  |
| Wedge | 6.90 | 9.36 | 0.03 | 0.461 | -11.48, 25.28 |  |  |  |  |  |
| Intraoperative bleeding volume | -0.05 | 0.04 | -0.04 | 0.230 | -0.12, 0.02 |  |  |  |  |  |
| Surgical duration | -0.04 | 0.08 | -0.02 | 0.559 | -0.19, 0.10 |  |  |  |  |  |
| Number of mediastinal lymph nodes retrieved | 0.36 | 0.81 | 0.02 | 0.657 | -1.23, 1.95 |  |  |  |  |  |
| Mediastinal lymph node stations explored | -0.54 | 2.30 | -0.01 | 0.815 | -5.04, 3.97 |  |  |  |  |  |
| Preoperative ALB | -2.51 | 0.97 | -0.09 | 0.010 | -4.41, -0.60 | -1.55 | 0.97 | -0.06 | 0.112 | -3.46, 0.36 |
| Preoperative D-Dimer | 3.82 | 5.63 | 0.02 | 0.498 | -7.23, 14.87 |  |  |  |  |  |
| Preoperative INR | 44.97 | 44.13 | 0.04 | 0.308 | -41.65, 131.60 |  |  |  |  |  |
| Preoperative APTT | -1.37 | 1.18 | -0.04 | 0.246 | -3.69, 0.95 |  |  |  |  |  |
| Preoperative TT | -6.77 | 1.69 | -0.14 | <0.001 | -10.08, -3.45 | -2.26 | 1.77 | -0.05 | 0.203 | -5.74, 1.22 |
| Preoperative PT | 4.99 | 4.06 | 0.04 | 0.219 | -2.97, 12.95 |  |  |  |  |  |
| Preoperative FIB | 0.40 | 0.05 | 0.26 | <0.001 | 0.29, 0.50 | 0.32 | 0.06 | 0.21 | <0.001 | 0.21, 0.43 |
| APTT, activated partial thromboplastin time; BMI, body mass index; CI, confidence interval; FIB, fibrinogen; HBA, hemocoagulase bothrops atrox; IPTW, inverse probability of treatment weighting; INR, international normalized ratio; M(P25,P75), median(25th percentile,75th percentile); M-VATS, multiportal video-assisted thoracoscopic surgery; PT, prothrombin time; SE, standard error; TT, thrombin time; TNM stage, Tumor, Node, and Metastasis stage; U-VATS, uniportal video-assisted thoracoscopic surgery; VATS, video-assisted thoracoscopic surgery. | | | | | | | | | | |
